# Reciprocal Activation of Transcription Factors Underlies the Dichotomy between Proliferation and Invasion of Glioma Cells

**DOI:** 10.1371/journal.pone.0072134

**Published:** 2013-08-15

**Authors:** Harshil D. Dhruv, Wendy S. McDonough Winslow, Brock Armstrong, Serdar Tuncali, Jenny Eschbacher, Kerri Kislin, Joseph C. Loftus, Nhan L. Tran, Michael E. Berens

**Affiliations:** 1 Translational Genomics Research Institute, Phoenix, Arizona, United States of America; 2 St. Joseph’s Hospital and Medical Center, Phoenix, Arizona, United States of America; 3 Mayo Clinic Arizona, Scottsdale, Arizona, United States of America; Cleveland Clinic, United States of America

## Abstract

Histology of malignant glioma depicts dense proliferative areas rich in angiogenesis as well as dissemination of neoplastic cells into adjacent brain tissue. Although the mechanisms that trigger transition from proliferative to invasive phenotypes are complex, the dichotomy of cell proliferation and migration, the “Go or Grow” hypothesis, argues for specific and coordinated regulation of these phenotypes. We investigated transcriptional elements that accompany the phenotypes of migration and proliferation, and consider the therapeutic significance of the “Go or Grow” hypothesis. Interrogation of matched core and rim regions from human glioblastoma biopsy specimens *in situ* (n = 44) revealed higher proliferation (Ki67 labeling index) in cells residing at the core compared to the rim. Profiling activated transcription factors in a panel of migration-activated versus migration-restricted GBM cells portrayed strong NF-κB activity in the migratory cell population. In contrast, increased c-Myc activity was found in migration-restricted proliferative cells. Validation of transcriptional activity by NF-κB- or c-Myc-driven GFP or RFP, respectively, showed an increased NF-κB activity in the active migrating cells, whereas the proliferative, migration restricted cells displayed increased c-Myc activity. Immunohistochemistry on clinical specimens validated a robust phosphorylated c-Myc staining in tumor cells at the core, whereas increased phosphorylated NF-κB staining was detected in the invasive tumor cells at the rim. Functional genomics revealed that depletion of c-Myc expression by siRNA oligonucleotides reduced cell proliferation *in vitro*, but surprisingly, cell migration was enhanced significantly. Conversely, inhibition of NF-κB by pharmacological inhibitors, SN50 or BAY-11, decreased both cell migration *in vitro* and invasion *ex vivo*. Notably, inhibition of NF-κB was found to have no effect on the proliferation rate of glioma cells. These findings suggest that the reciprocal and coordinated suppression/activation of transcription factors, such as c-Myc and NF-κB may underlie the shift of glioma cells from a “growing-to-going” phenotype.

## Introduction

Glioblastoma multiforme (GBM) is the most common and most lethal primary malignant brain tumor, affecting 25,000 patients per year [1]. Despite major research efforts and advances in diagnosis and treatment, overall survival of patients has improved little over the last 30 years [2] and remains at a mean of 14.6 months [3]. Two major aspects of glioma biology that contribute to its poor prognosis are microvascular proliferation and diffuse infiltration of glioma cells [4]. Invasion of normal brain by infiltrating tumor cells makes complete surgical removal of the tumor very challenging and underlies therapeutic failures [5]. To date, no specific treatment has been developed targeting this lethal tumor cell phenotype [6]–[8]. Invading GBM cells have been posited to include disproportionate fractions of tumor progenitor cells in proliferative dormancy [9], [10].

Past studies have attempted to discern genomic differences between glioma cells at the stationary tumor core and the invading peritumoral rim. *In vitro* experiments with glioma cells demonstrated that cell migration and proliferation are stochastically mutually exclusive processes and that glioma cells defer cell division in order to migrate and vice versa [11], [12]. Observations reported by Hatzikirou *et al.* further supported this phenomenon of migration or proliferation (also known as “Go or Grow” mechanism) using a mathematical model to better describe both recurrence patterns and the rates of recurrent GBM [13]. Moreover, transcriptional profiling of laser capture-microdissected cells collected from paired patient GBM tumor core and invading rim (n = 19) revealed distinct gene signatures, which have been confirmed both at the mRNA and protein levels [14]. Importantly, this invasive gene signature displays some overlap with the differential expression patterns of the migrating gene signature *in vitro* [15]. Together, these studies identified candidate genes potentially involved in cell migration or invasion, as well as genes involved in anti-apoptotic (survival) related functions upregulated in invasive (rim) cells. Additionally, migrating/invading glioma cells display a lower proliferative index as compared to stationary glioma cells by suppressing entry into the cell cycle [12]. This altered gene expression accompanied by the change in phenotypes are likely due to transcriptional regulation [16] which may confer their spatially restricted behavior. Thus, identifying specific transcription factors that are master regulators of specific glioma cell signatures [17], proliferative versus invading, can lead to better understanding progression of this disease, and identify novel therapeutic targets specific to the invasive phenotype.

Here, we investigate transcription factors that are potential regulators of certain GBM cell phenotypes. We report clinically validated mechanistic findings that glioma cells residing at the tumor core have a heightened commitment to proliferate and that migrating/invading cells at the tumor rim demonstrate a low proliferative index. Increased NF-κB activity was observed in migrating/invading cells, whereas, c-Myc activation was detected in migration-restricted, proliferative cells at the tumor core. Interestingly, depletion of c-Myc expression by siRNA oligonucleotides not only suppresses glioma cell proliferation but enhances cellular migration. In contrast, inhibition of NF-κB function suppresses cell migration but has no effect on cell proliferation. Thus, our data support the notion that a switch between cell proliferation and cell migration is likely dictated by the temporal activation/suppression of certain transcription factors.

## Materials and Methods

### Expression Profile Dataset of NF-κB and c-Myc Target Genes in Human Gliomas

The expression microarray database of laser capture microdissected GBM cells collected from 19 paired patient GBM tumor core and invading rim (GES12689) was previously described [18]. For our analysis, gene expression data were normalized in two ways: per chip normalization and per gene normalization across all samples in the collection. For per chip normalization, all expression data on a chip were normalized to the 50^th^ percentile of all values on that chip. For per gene normalization, the data for a given gene were normalized to the medium expression level of that gene across all samples. Gene expression differences were deemed statistically significant using parametric tests where variances were not assumed equal (Welch ANOVA). Expression values were then filtered for highly variable (differentially expressed) genes (coefficient of variation, >30%) across matched rim/core samples.

### Invasion Tissue Microarray (TMA)

A TMA containing representative punches of tumor core, edge, and invasive rim from 44 clinically annotated cases of WHO grade IV GBM specimens (according to standardized criteria [19]) from 10 institutes was previously described [20]. The glioma samples were obtained from patients who underwent primary therapeutic subtotal or total tumor resection performed under image guidance. All specimens were collected and submitted to the study under institutional review board–approved protocols. The formalin-fixed paraffin-embedded tissue blocks were selected by both a neurosurgeon and a neuropathologist from each institution. In addition, each specimen block chosen met the criteria of non-necrotic, non-irradiated, or chemo-treated glioma tissue. Briefly, two separate face cuts were made for each of the specimens used to construct the TMA. H&E staining was performed on the face cuts to assist in the identification of the tumor cells. Two neuropathologists (Dr. Ken Aldape, MD Anderson, and Dr. David Zagzag, New York University) independently reviewed the face cuts and designated the areas of core (center of the tumor) and rim (region distal to the edge but still containing notable tumor cells). The TMA was constructed from representative punches of tumor core and invasive rim using an indexed manual arrayer with attached stereomicroscope (Dr. Galen Hostetter, pathologist and Director of the TMA core facility at Translational Genomics Research Institute). Tissue microarray slides were stained by H&E every 50 sections to confirm the presence of tumor, and that tissue morphology and diagnosis were consistent.

### Immunohistochemistry–Fluorescence Staining

Five-micrometer thick sections from the glioma invasion tissue microarray (TMA) were baked at 65°C for 1 hour, deparaffinized in three xylene washes, followed by a dehydration series in graded ethanol, then rehydrated in water. The slide was blocked, and antigens were retrieved using a sodium citrate based buffer, pH 6.5, for 20 minutes (BondMax autostainer; Vision Biosystems, Norwell, MA). For immunohistochemistry (IHC) staining, a primary rabbit polyclonal antibody, anti-Ki67 (MIB-1) (CP249B, Biocare, Concord, CA), was diluted 1∶100 and incubated for 30 minutes. Following incubation and rinsing of the primary antibodies, slides were incubated with a secondary antibody conjugated to HRP for 30 minutes followed by a diaminobenzidine (DAB) substrate then counterstained with hematoxylin and coverslipped. For fluorescence staining, a primary rabbit polyclonal antibody, anti-Ki67 (MIB-1) (CP249B, Biocare, Concord, CA), was diluted 1∶100 and incubated for 30 minutes and a 1∶50 dilution of Alexaflour® 488 conjugated mouse anti–glial fibrillary acidic protein (GFAP) (#3655 Cell Signaling Technology, Boston, MA) was used to stain the cytoplasmic portions of glial cells. A secondary antibody cocktail containing anti-rabbit Alexa 555 was incubated for 30 minutes. Lastly, a tyramide signal amplification system (Perkin Elmer, Waltham, MA) was used to increase the sensitivity of fluorescence detection. The slide was coverslipped using ProLong Gold containing 4′,6-diamidino-2-phenylindole (DAPI) (Invitrogen).

IHC staining for phospho c-Myc (#9401 Cell Signaling Technology, Boston, MA), total c-Myc (#9402 Cell Signaling Technology, Boston, MA), phospho NF-κB (#3037 Cell Signaling Technology, Boston, MA)and total NF-κB (#4764 Cell Signaling Technology, Boston, MA) was performed. Following incubation and rinsing of the primary antibodies, slides were incubated with a HRP-conjugated secondary antibody for 30 minutes followed by a DAB substrate; sections were counterstained with hematoxylin then coverslipped.

### Image Acquisition and Analysis

Fluorescence-based automated and quantitative analysis (AQUA; HistoRx technology [18], [21]) measured alterations in the levels of expression of Ki67 within regions of interest defined by GFAP immunoreactivity within each spot of the TMA. The AQUA score is a numeric representation of fluorescence intensity in a user-defined area normalized by exposure time. Multiple, monochromatic, high resolution (2048 × 2048 pixels) images were obtained from each TMA spot with an Olympus BX51RF microscope (Olympus, Melville, NY) [18] and analyzed with the AQUA software. For each TMA spot, areas of tumor are distinguished from stromal elements by creating a region-of-interest mask based on GFAP signal (Alexa 488 fluorophore). Positivity for GFAP was set by gating the pixels in which an intensity threshold was set by visual inspection of TMA spots, and each pixel was recorded as “tumor” or “nontumor” by the software on the basis of the threshold. The DAPI image, used to designate the nuclei, was subjected to a rapid exponential subtraction algorithm that improves signal-to-noise ratio by subtracting the out-of-focus image from the in-focus image. After applying the exponential subtraction algorithm, the signal intensity of the Ki67 target protein (Alexafluor® 555 signal), was scored on a scale of 0 to 255. The AQUA score within the subcellular compartment of the cytoplasm was calculated by dividing the signal intensity by the area of the specified cytoplasmic compartment. Separate manual scoring of Ki67 IHC (by a pathologist J.M.E.) on the TMA was performed using a system for chromophore to capture the outcome: 0, negative; 1, very weak; 2, moderate; 3, intense staining. Scoring for phospho c-Myc, total c-Myc, phospho NF-kB, and total NF-kB levels on the TMA was also performed using a system for chromophore to capture outcome (0, negative; 1, very weak; 2, moderate; 3, intense staining) by a pathologist (J.M.E.).

### Cell Culture and Cell Lines

Human glioma cell lines SF767 [22], U87, T98G, and SNB19 (American Type Culture Collection, Manassas, VA) were maintained in DMEM with high glucose (Invitrogen, Carlsbad, CA) supplemented with 10% heat-inactivated fetal bovine serum (Invitrogen, Carlsbad, CA).

### Transcription Factor (TF) Analysis using Luminex

SNB19, T98G, SF767, and U87 glioma cells were seeded on glioma-derived ECM [22], [23] under migration-activated (sparse cell seeding: 3×10^5^/100 mm dish) or migration-restricted (dense cell seeding: 5×10^6^/100 mm dish) conditions. After 24 hrs, nuclear extracts were collected (Marligen’s Nuclear Extraction Kit, Cat# 11906-100); biological replicate samples were collected in duplicate. Protein concentration was determined, and adjusted, based on a BCA kit (Pierce, Rockford, IL). Samples were run in triplicate as per Marligen’s Multiplex Transcription Factor Profiling Kit (19-plex). Median fluorescent intensities (MFI) for triplicate wells were averaged. MFI readouts comparing the activation state of each of the 19 TFs consequent to migration (as a ratio migration-activated v migration-restricted input for each cell line) were analyzed using a generalized linear model with contrasts (to overcome uncertainty as to the normal distribution of the data; to overcome a restricted mean of the ratio data of MFIs; and to accommodate the likelihood that the variance of the data is not constant for all observations) [24]–[26]. Statistical significance was assigned if p<0.003 to accommodate Bonferoni correction to assure minimal false-discovery rates from small sample sizes.

### siRNA Preparation and Transfection

Sets of siRNA oligonucleotide specific for GL2 luciferase [27] and for c-Myc were designed, validated, and purchased from Qiagen (Valencia, CA): c-Myc-5 (5′ GAT CCC GGA GTT GGA AAA CAA) and c-Myc-7 (5′ CTC GGT GCA GCC GTA TTT CTA). For positive cell death by siRNA transduction, AllStars Hs Cell death Control siRNA™ (Qiagen, Valencia, CA) was used according to manufacturer’s guidelines. Cells were plated in a 60-mm plate at 8.0 × 10^5^ cells per plate in 3 ml of Dulbecco’s modified Eagle’s medium, supplemented with 10% serum without antibiotics. After cell attachment, transfections with c-Myc siRNA oligonucleotides or control GL2 luciferase siRNA oligonucleotides were carried out according to the manufacturer’s protocol (20 nM for 16 hours). Cells were assayed on either day 1 or day 2 after transfection. No cell toxicity was observed using this protocol.

### Western Blot Analysis

Immunoblot analysis and protein determination experiments were performed as previously described [28]. Briefly, monolayers of cells were washed in phosphate-buffered saline (PBS) containing 1 mM phenylmethylsulfonyl fluoride (PMSF) and then lysed in 2× sodium dodecyl sulfate (SDS) sample buffer (0.25 M Tris-HCl, pH 6.8, 2% SDS, and 25% glycerol) containing 10 µg/ml aprotinin, 10 µg/ml leupeptin, and 1 mM PMSF. Protein concentrations were determined using the BCA assay procedure (Pierce, Rockford, IL), with bovine serum albumin as reference. Fifteen micrograms of total cellular protein was loaded per lane, separated by 10% SDS–polyacrylamide gel electrophoresis, and then transferred to nitrocellulose (Invitrogen) by electroblotting. The nitrocellulose membrane was blocked with 3% BSA in Tris-buffered saline (pH 8.0) with 0.1% Tween-20 before the addition of primary antibodies: c-Myc (#9402, 1∶1000, Cell Signaling, Boston, MA); and NF-κB (#4764, 1∶1000, Cell Signaling, Boston, MA). HRP conjugated anti–rabbit IgG was diluted 1∶2000 (Promega, San Luis Obispo, CA). Bound secondary antibodies were detected using a chemiluminescence system (NEN Life Science Products, Boston, MA). A mouse monoclonal antibody for α-tubulin clone DM1A (Millipore) was used to normalize transferred protein.

### Cell Proliferation Assay

The Alamar Blue assay (Biosource, Camarillo, CA) was used to assess cell proliferation as described previously [29]. Briefly, 750 GBM cells were seeded (8 replicate wells per condition) in 96-well plates in 100 µl of DMEM supplemented with 10% fetal bovine serum and allowed to attach at 37° for 6 hrs. Luciferase (control) or c-Myc siRNA complexes were diluted into DMEM supplemented with 10% FBS and added to the respective wells. After 24, 48, 72 and 96 hrs Alamar Blue was added to the cells in a volume of 10 µl (10% of total volume) and incubated at 37°C for 4 hrs. A standard curve using an 8-point serial dilution of cells (beginning with 8,000 cells/well) was prepared. The plates were read on a fluorescence plate reader (Biotek Synergy HT) excitation 560 nm; emission 600 nm. Averages of the fluorescence values were calculated and the cell numbers were determined from the standard curve.

### Radial Cell Migration Assay and BrdU Incorporation

Radial dispersion of cells was determined using a microliter scale migration assay as described previously [28]. Briefly, 10-well slides were coated with 10 µg/ml human laminin at 37°C for 1 hour and washed with PBS to enhance cell attachment while promoting migration. Approximately 3000 cells were deposited as a 1 mm confluent disc of cells using a cell sedimentation manifold (CSM, Inc., Phoenix, AZ). For Bromodeoxyuridine (BrdU)-incorporation, cells were treated in monolayer at a final BrdU concentration of 10 µM for 2 hours prior to sedimentation using manifolds. After sedimentation and adhesion, cells were allowed to disperse for 48 hours, then fixed (4% paraformaldehyde) and permeabilized (0.1% Triton X-100). After washing with PBS and blocking (1% BSA +3% goat serum), cells were incubated with primary antibodies, rabbit anti-Cyclin A (Santa Cruz Biotechnology, Santa Cruz, CA) and mouse anti-BrdU (#5292 Cell Signaling Technology, Boston, MA) for 1 hr at 25°C. Following washing, cells were incubated with secondary antibodies, Cy3 conjugated anti-rabbit IgG (Zymed, San Francisco, CA), FITC conjugated anti-mouse IgG (BD Bioscience, San Jose, CA), and with DAPI as a nuclear counterstain. Epifluorescence was examined by laser scanning confocal microscopy (LSM 510, Zeiss). The percent of Cyclin A-, and BrdU-labeled cells, and the total number of cells were measured at the core and the rim of the radial migration assays.

For cell migration after siRNA transfection, cells were transfected with siRNA in monolayer before transferring into manifolds. After sedimentation and adhesion, cells were allowed to disperse for 24 to 48 hours. Measurements of the area occupied by the cells were taken at regular intervals for 48 hours. The average radial migration rate of five replicates was calculated as the increasing radius of the entire cell population over time [30].

For cell migration after NF-κB inhibitor treatment, cells were deposited on 10-well slides using cell sedimentation manifold. Once adherent, cells were treated with 50 µM SN50 (NF-κB inhibitor peptide) (Calbiochem, Gibbstown, NJ, cat # 481480) or 50 µM SN50M (scrambled peptide) (Calbiochem,Gibbstown, NJ, cat #481486) or 20 µM BAY-11-7082 (small molecule inhibitor of NF-kB) (Cayman Chemical Company, Ann Arbor, MI) or DMSO (vehicle control) or left untreated. After NF-kB inhibitor treatment, cells were allowed to disperse for 24 to 48 hours. Measurements of the area occupied by the cells were taken at regular intervals for 48 hours. The average radial migration rate of five replicates was calculated as the increasing radius of the entire cell population over time.

### Organotypic Brain Slice Invasion Assay

An *ex vivo* invasion assay into rat brain slices was done as described previously [31]. Briefly, 400-µm-thick vital sections were prepared from brains of Wistar rats (Charles River Laboratories) floated on micropore membranes in culture media. Glioma cells (1 X 10^5^; T98G and SNB19) stably expressing green fluorescence protein (GFP) were gently deposited (0.5-µL transfer volume) bilaterally onto the putamen of the rat brain slice. Deposited cells were then treated with SN50, SN50M, BAY-11-7082, or left untreated (NT) and observed at 48 hours. Forty-eight hours after seeding the cells, glioma cell invasion into the rat brain slices was detected using a LSM 5 Pascal laser-scanning confocal microscope (Zeiss) to observe GFP labeled cells in the tissue slice. Serial optical sections were obtained every 10 µm downward from the surface plane to the bottom of the slice, and for each focal plane, the area of fluorescent cells as a function of the distance from the top surface of the slice was calculated. The extent of glioma cell invasion was reported as the depth where the area of fluorescent tumor cells was half of the maximum area at the surface.

### NF-κB-GFP and c-Myc-tdTomato Reporter Constructs

NF-κB-GFP based reporter lentiviral vector (pGreenFire1–NF-κB, Catalog # TR012PA-1) and control-GFP vector (pGreenFire1–mCMV, Catalog # TR010PA-N) were purchased from SBI (Mountain view, CA). c-Myc-tdTomato based reporter lentiviral vector was constructed using the following method. A PCR product of 122 nt, containing six tandem copies of the c-Myc-binding E-box sequence (CACGTG) upstream of the herpes simplex virus thymidine kinase promoter TATA box, was made with the primers CGG CTG TTC GAA GGT ACC GAG CTC TTA CGC GTG C and GTC GCG GGA TCC ATA CCC AGA TCT CAC GTG CA, using pMyc-TA-Luc as a template (Clontech Laboratories, Mountain View, CA). The PCR amplicon was digested with Bst BI and Bam HI (restriction sites underlined in primer sequences), and cloned in the Bst BI and Bam HI sites of the promoterless pLVX-DD-tdTomato vector, encoding a tdTomato protein with proteotuner domain (Clontech Laboratories, Mountain View, CA). The final pLVX-MYC-DD-tdTomato reporter plasmid was verified by sequencing. Standard molecular biology methods were used for plasmid preparation and propagation. pLVX-DD-tdTomato vector with P_CMV IE_ (human cytomegalovirus immediate early promoter) promoter element (Clontech Laboratories, Mountain View, CA) was used as control.

Recombinant lentiviruses were produced as described elsewhere [32], [33]. Briefly, 293T cells were transiently transfected with 20 µg of the appropriate lentiviral transfer vector construct, 15 µg of psPAX2 packaging plasmid, and 5 µg of pMD2G-VSVG envelope vector by calcium phosphate precipitation. Recombinant lentivirus containing supernatants were harvested 48 h after transfection. For lentiviral transduction, medium containing appropriate recombinant lentiviruses was added to subconfluent cultures of cells (SNB19 and T98G). Forty-eight hours after infection, cells were harvested and used for further analysis.

### NF-κB-GFP and c-Myc-tdTomato Reporter Assay

NF-κB-GFP and c-Myc-tdTomato reporter assays were performed using radial migration experimental setup. Briefly, approximately 3000 cells were deposited as a 1 mm confluent disc of cells using a cell sedimentation manifold on 10 µg/mL laminin coated 10-well slides. After sedimentation and adhesion, cells were allowed to disperse for 48 hours. Cells were then stained with Hoecsht 33258 (Invitrogen, Carlsbad, CA) as a nuclear counterstain. Epifluorescence was examined by laser scanning confocal microscopy (LSM 510). The percent of GFP or RFP (tdTomato) positive cells, and the total number of cells at the core and rim region were measured. For analyzing c-Myc reporter cell lines a proteotuner ligand (Shield®, Clontech Laboratories, Mountain View, CA) at a final concentration of 500 nM was used to stabilize rapidly degrading tdTomato protein and enhance RFP signal during fluorescence imaging.

### Ethics Statement

Human biospecimens used in this study are pre-existing and de-identified. Translational Genomics Research Institute (TGen) investigators will not have access to patient identifiers at any time before or after completion of the study. TGen investigators and the holder of the key will enter into an agreement prohibiting the release of the key to TGen investigators under any circumstances. Therefore, the biospecimens do not qualify as human subjects, in accordance with OHRPs “Guidance on Research Involving Coded Private Information or Biological Specimens”.

## Results

### Ki-67 mRNA Expression is Higher in the Core of GBM Tumors than in Matched Rim Regions

**Figure 1:**
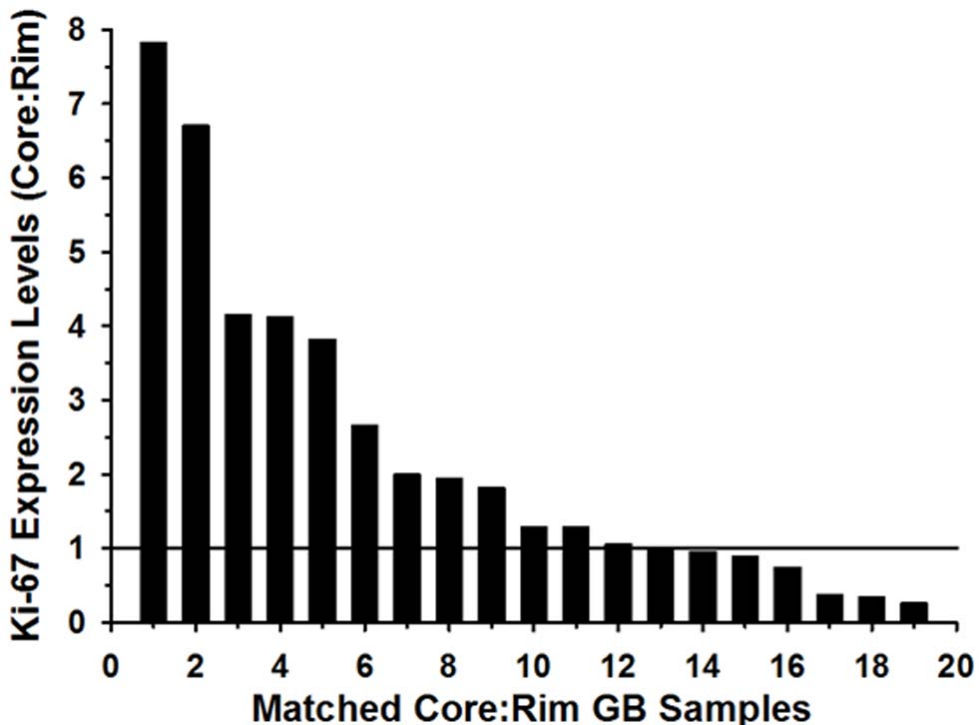
Ki-67 is overexpressed in GBM cells at the tumor core. From 19 independent GBM specimens, 1000 to 2000 stationary (core) and invasive (rim) cells were harvested by LCM for microarray analysis. Relative Ki-67 mRNA signal intensity was expressed as a ratio of rim to core.

**Figure 2:**
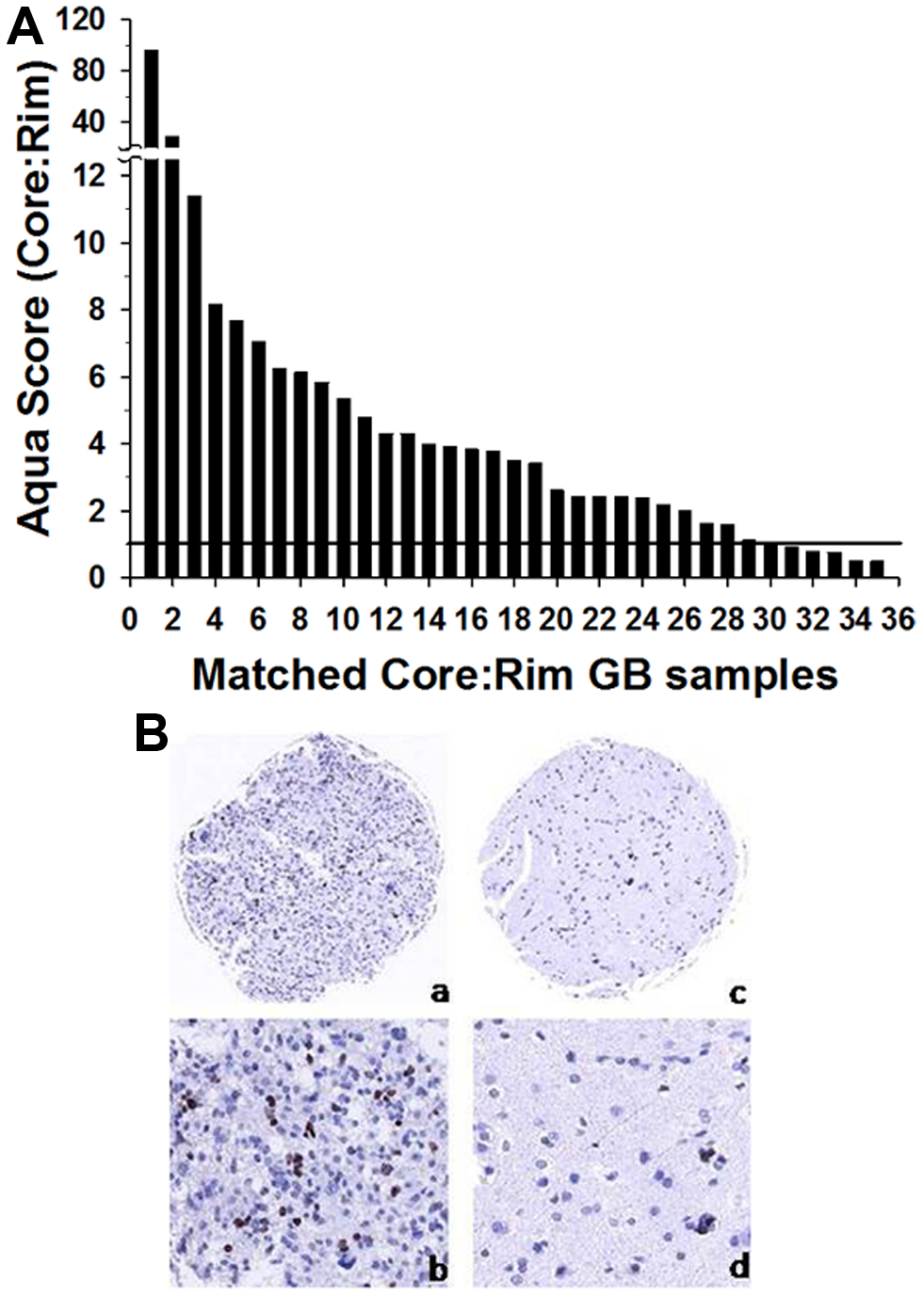
Quantitative analysis of Ki-67 expression in 35 human glioma tumors. (A) AQUA scores of Ki-67 protein levels were measured in matched sets of core:rim for each tumor; ratio of AQUA scores for each specimen are plotted, ranked from high to low. Samples with an AQUA score fold difference above “1” indicates significant up-regulation (indicated by horizontal line) (p<0.002). (B) Representative images of the Ki-67 (MIB-1) immunostaining of the GBM core (a, b) and invasive rim (c, d). Original magnification 10x (a,c) and 20x (b,d).

We previously showed that migrating cells *in vitro* display a lower proliferation index than migration-restricted cells (12). To determine the clinical significance of the dichotomy of GBM cell migration and proliferation, we examined Ki-67 mRNA expression in the GBM invasion expression arrays from a panel of 19 GBM specimens. In 11 of 19 glioblastoma matched core and rim biopsies, the mRNA expression of Ki-67 at the core is 1.2 to 7.8– fold elevated compared with matched samples of cells invading at the rim (Figure 1) (pairwise t-test, p<0.05). Additionally, we examined the protein expression of Ki-67 on an invasion glioma tissue microarray consisting of 44 GBM cases assembled to reflect the dispersion of infiltrative gliomas. Quantitative AQUA scores [18] of Ki-67 IHC staining within GFAP regions of interest were derived for matched samples (for representative fluorescence micrographs of matched core and rim regions see Figure S1). Ratios of AQUA scores of Core/Rim >1 were observed in 29 of 35 cases (only 35 analyzable matched core and rim specimen out of total 44 specimen on TMA) (Figure 2A). To determine the proliferation indices, the Ki67-positive tumor cells were scored and divided by total tumor cells in high powered fields (n = 200 cells). The proliferation indices of the GBM core samples ranged from 0.5 to 56% with a median value of 6; the proliferation indices of the GBM rim samples ranged from 0 to 20% with a median value of 3. Higher proliferative indices were observed in the core compared with the invasive rim in 29 of 35 cases, corroborating the mRNA expression level. Pairwise t-test indicates an increase in cell proliferation in the core versus the rim (p<0.002). These results suggest that invading glioma cells *in situ* may suppress cell proliferation.

### Radial Dispersion of Glioma Cells *in vitro* is Accompanied by a Deferred or Suppressed Entry into Cell Cycle

**Figure 3:**
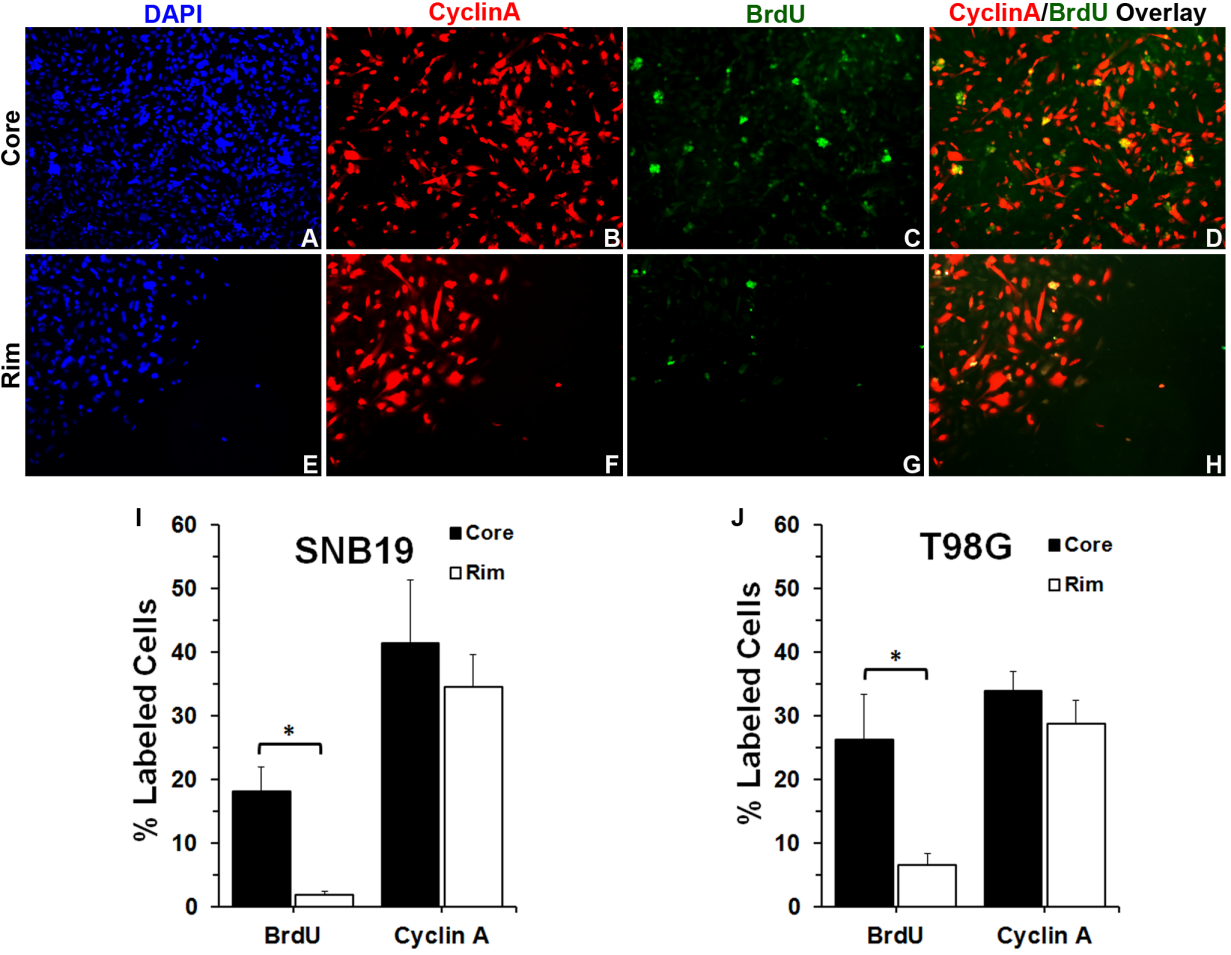
Glioma cells seeded in a way that manifests cell crowding and cell dispersion show that at the core cells were more proliferative than glioma cells located at the rim. (A) SNB19 cells at the core of the cell circle stained for DAPI to account for all cells. (B) Same image field as panel A at the core of the cell circle but stained for Cyclin A (Cy3-red), (C) incorporated BrdU (FITC-green), and (D) showing CyclinA (Cy3-Red) - BrdU (FITC-green) Overlay. (E) SNB19 cells at the rim of the cell circle stained for DAPI to account for all cells. (F) Same image field as panel E but stained for Cyclin A (Cy3-red), (G) incorporated BrdU (FITC-green), and (H) showing CyclinA (Cy3-Red) - BrdU (FITC-green) Overlay. (I) and (J) Quantitative analysis of Cyclin A- and BrdU-labeled SNB19 and T98G glioma cell numbers (n = 3). The percent of Cyclin A-, and BrdU-labeled cells, and the total number of cells were measured. Statistical analysis was performed using a student T-test. * p<0.05.

Cells which were either stationary or migrating were assessed for engagement in cell cycle (either DNA synthesis or Cyclin A expression). Glioma cells were tagged as being in cell cycle using BrdU incorporation immediately prior to seeding in the radial migration assay. Cells in active cell cycle 48 hours after seeding in the radial migration assay were detected by staining for Cyclin A (cell is in either S or G_2_ phase of cell cycle [34]–[36]). At the end of 48 hr, cells were fixed, stained, and then imaged under laser scanning microscope. Representative fluorescent images of the core and the rim regions of stained SNB19 cells (green, BrdU positive; red, Cyclin A positive; blue, nucleus) are shown in Figure 3(A–H and Figure S2). The percent of Cyclin A-positive cells and BrdU-labeled cells out of the total number of cells for core and rim regions were measured (Figure 3 I–J). In the core region, 18% of SNB19 and 26% of T98G cells evidenced BrdU incorporation, however very few cells (2% SNB19 and 7% T98G cells) that had incorporated BrdU were detected at the rim of the seeded region (Figure 3 I–J). By Cyclin A expression, nearly equivalent fractions of cells were measured in the core and the rim regions. This assessment of which cells migrate on deposition onto pro-motility substrates, suggests that cells that had not been proliferating when encountering substrate migrated to the rim preferentially to cells that had been in cell cycle immediately before substrate adhesion and that cells which had been in cell cycle were less likely to adopt migratory activity; the Cyclin A staining supports a conclusion that cells at the core and at the rim are equally able to enter cell cycle. An interpretation consistent with these observations would be that actively proliferating glioma cells defer migration. These results coincide with our previous data showing that glioma cells delay active proliferation to migrate, while stationary cells have an increased tendency to proliferate [12], suggesting that cell proliferation and migration may be interrelated but dichotomous behaviors.

### Transcription Factor Profiling of Stationary versus Migrating Glioma Cells

**Figure 4:**
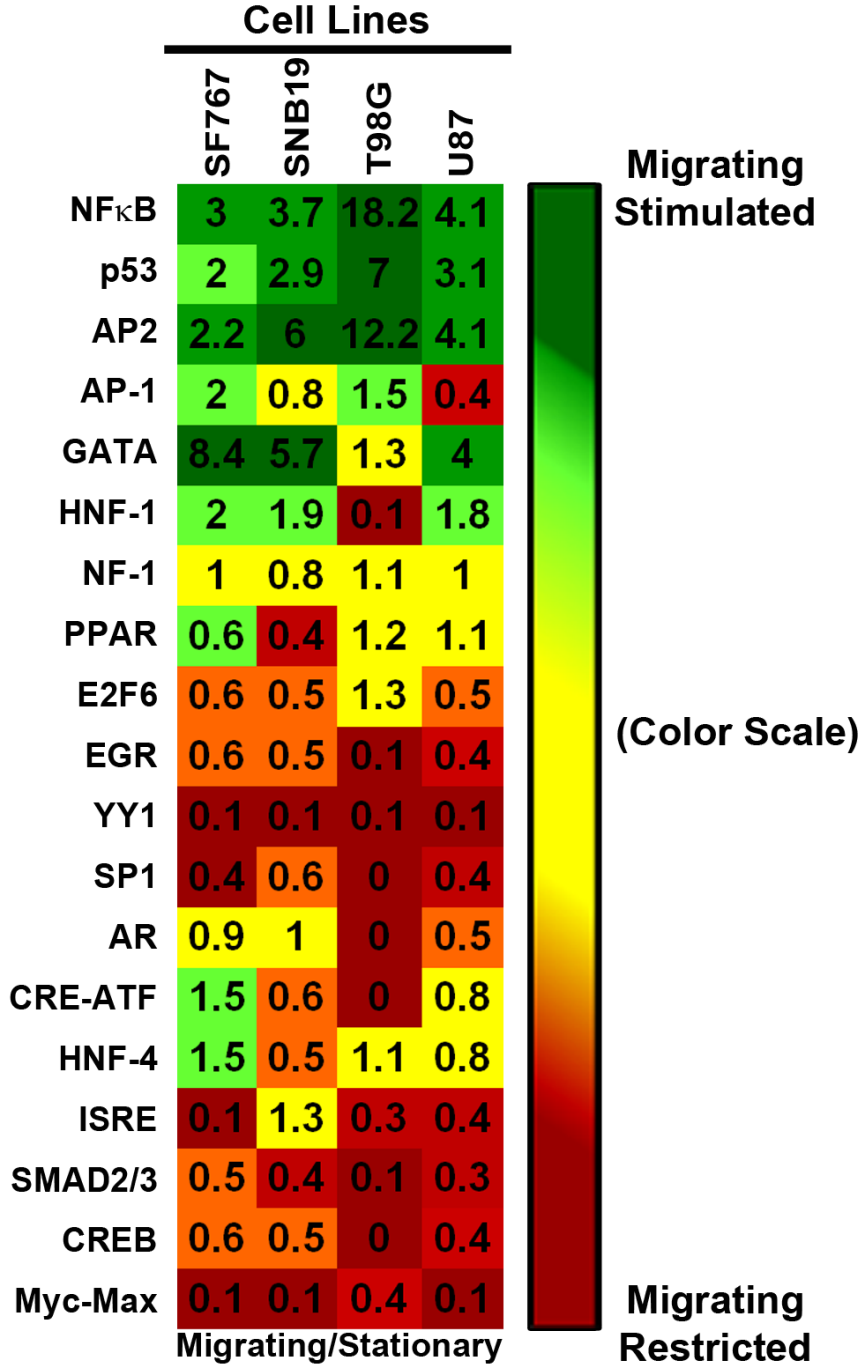
Transcription Factor Profiling of Migrating Glioma Cells vs. **Migration-Restricted Glioma Cells.** Tumor cells were seeded in either migration-activated “sparse” condition or in migration-restricted “dense” condition on SF767 glioma-derived ECM. Nuclear lysates were collected and hybridized with biotinylated-DNA probes specific to 19 transcription factors as per Marligen’s transcription kit and assayed using the Luminex 200. Two independent biological replicates were performed with each sample in triplicate. Ratios of the averaged mean fluorescent intensities for each transcription factor for sparse over dense were calculated for each biological set and average values of two biological replicates are plotted in the heat map above (For ratios of the average MFI for each biological replicate is presented in Figure S2). The heat map was constructed using a conditionally formatted color range. Green boxes represent the transcription factors activated when cells were in a migration-activated condition (sparse/dense ratios ≥1.5). Red boxes represent transcription factors activated when cells were in a migration-restricted condition (sparse/dense ratios ≤0.6). Yellow boxes indicate no change in transcription activity (sparse/dense ratios between 0.65 and 1.5).

To determine changes in transcriptional regulation between stationary glioma cells at the tumor core and invading glioma cells at peritumoral rim, we compared the activity of 19 transcription factors in migration-activated glioma cells versus migration-restricted glioma cells. Activated TFs were detected using specifically designed probes that contain TF binding sequence present at promoter regions of genes that are regulated by that TF. Ratios of MFI values of activated TFs in migration-activated versus migration-restricted cells are color-coded with respect to magnitude of change and are presented in the form of a heat map (Figure 4). Across all 4 glioma cell lines, NF-κB, p53, and AP2 are the most strongly activated TFs coincident to migration, whereas low activation of CREB and Myc are observed in migrating tumor cells as compared to stationary, core tumor cells. Further, similar patterns of the transcription factor activation profile was observed across a panel of breast cancer, pancreatic cancer, and melanoma cell lines (Figure S3) when assayed in migration-restricted vs migration-activated conditions, suggesting a conserved signaling mechanism for tumor cell migration irrespective of cell origin.

### Immunohistochemical Validation of c-Myc and NF-κB Activation in a Glioma Invasion TMA

**Figure 5:**
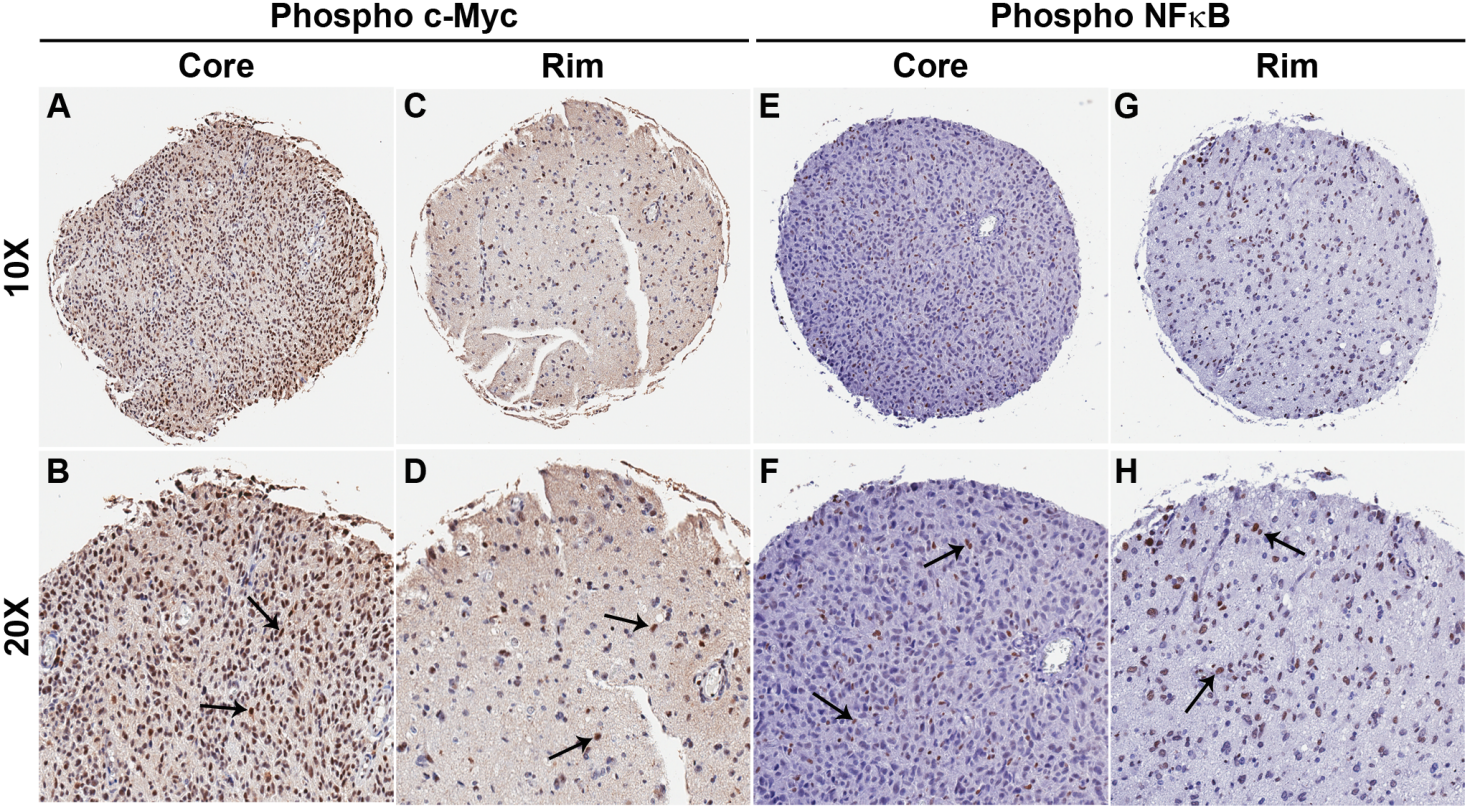
Glioma tumor specimens show differential activation of c-Myc and NF-κB in core and invasive rim. Immunohistochemistry of matched glioma core and rim sample from a glioma invasion specific tissue microarray (n = 45). Phosphorylated c-Myc nuclear protein expression is greater at the glioma tumor core than the rim regions of tumor. Representative 10X images of core (A) and rim (C) of a GBM sample. 20X images of core (B) and rim (D) of the same GBM sample. Phosphorylated NFκB nuclear protein expression is greater at the glioma tumor rim than the core regions of tumor. Representative 10X images of core (E) and rim (G) of a GBM sample. 20X images of core (F) and rim (H) of the same GBM sample. Black arrows represent positively stained nuclear regions of the glioma tumor cells.

Because c-Myc and NFkB are among the top reciprocal transcription factors in the migrating population, we assessed the clinical validation of the activation status of these transcription factors by measuring the levels of activated c-Myc (phospho c-Myc [37], [38]) and activated NF-κB (phospho p65/NF-κB [39]) on the invasion GBM TMA. IHC staining was analyzed by visual scoring and comparing the scores of matched core and rim specimens. Numbers of samples overexpressing activated c-Myc and NF-κB in matched core and rim specimens were quantified and are summarized in Table 1. Fisher’s exact t-test indicates that expression of activated c-Myc is significantly elevated in the tumor core as compared to rim whereas expression of activated NF-κB is higher in the rim as compared to core (p<0.001). Representative images of matched core and rim specimens are presented in Figure 5 (A–H and Figure S4). Analysis of level of total c-Myc (independent of phosphorylation status) in the core and rim regions demonstrated the same conclusion as using staining for phosphor c-Myc (Data not shown). These data demonstrate the levels of activation of transcription factors c-Myc and NF-κB in the two subpopulations of GBM cells with higher NFkB activation detected in the cells at the invading rim, and elevated c-Myc activation in cells residing in the tumor core *in situ*, corroborating the outcome of *in vitro* transcription factor profiling study.

**Table 1:** Summary of comparative analysis of IHC staining for activated c-Myc and NF-κB.

Target	High inCore	High inRim	NoChange	No tumorsample*
Phospho c-Myc	24	14	1	5
Phospho NF-κB	8	30	5	1

### Migrating Glioma Cells Promote Activation of the Transcription Factor NF-κB whereas Migration-restricted Glioma Cells Display High c-Myc Activation

**Figure 6:**
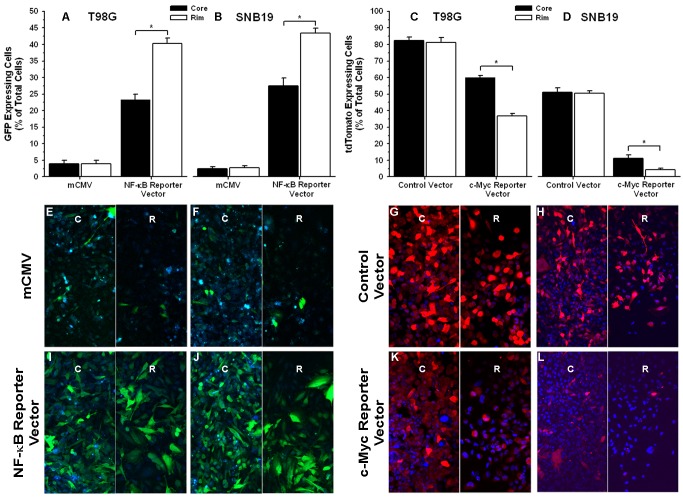
Migrating glioma cells promote activation of the transcription factor NF-κB whereas migration-restricted glioma cells display high c-Myc activation. T98G and SNB19 glioma cells were infected with lentivirus expressing the binding element for either the transcription factor NF-kB and a green fluorescent protein (GFP) reporter or the transcription factor c-Myc and a red fluorescent protein (tdTomato) reporter. Cells were plated in a migration stimulating environment and imaged after 48 hrs. (A) & (B) Quantitative analysis of GFP positive T98G and SNB19 glioma cells respectively, at the core and the rim in radial monolayer assay (n = 5). (C) & (D) Quantitative analysis of tdTomato positive T98G and SNB19 glioma cells respectively, at the core and the rim in radial monolayer assay (n = 5). (E) & (F) Fluorescent micrographs (20X) of mCMV control GFP vector infected T98G and SNB19 glioma cells respectively. (G) & (H) Fluorescent micrographs (20X) of control tdTomato vector infected T98G and SNB19 glioma cells respectively. (I) & (J) Fluorescent micrographs (20X) of NF-κB reporter vector infected T98G and SNB19 glioma cells respectively. (K) & (L) Fluorescent micrographs (20X) of tdTomato reporter vector infected T98G and SNB19 glioma cells respectively. Green cells are GFP positive and blue cells are not expressing the GFP protein but are stained with Hoescht stain. Red cells are tdTomato positive and blue cells are not expressing the tdTomato protein but are stained with Hoescht stain. Fluorescent micrographs of core regions are depicted by “C” and corresponding rim regions are depicted by “R”. Error bar represent the standard deviation of n = 5 observations. Asterisk (*) represents p value <0.05.

To functionally assess changes in the activation state of NF-κB and c-Myc in the two phenotypic subpopulations of GBM cells, we stably transduced T98G and SNB19 cells with NF-κB driven GFP or c-Myc-driven-tdTomato. Radial monolayer cell migration assay was performed on each reporter and control cell lines to assess the spatial activation (stationary core versus migrating rim) of NF-κB and c-Myc. Fluorescent micrographs of core and rim regions for reporter and control T98G and SNB19 glioma cells in a radial monolayer assay are presented in Figure 6 E–L (High magnification images are presented in Figure S5). Quantitative analysis of fluorescent micrographs representing NF-κB reporter and control glioma cell lines demonstrated a significant increase in percent of GFP positive cells (p<0.05) at the rim as compared to core suggesting higher activation of NF-κB in the migrating cells as compared to the stationary cells. Conversely, quantitative analysis of fluorescent micrographs representing c-Myc reporter and control glioma cell lines demonstrated a significant increase in the percent of tdTomato positive cells (p<0.05) at core as compared to rim suggesting higher activation of c-Myc in the stationary cells as compared to the migrating cells (Figure 6C & D). Cells transfected with control vector did not show any difference in percent GFP or tdTomato positive cells between core and rim region (Figure 6A & B).

### Knockdown of c-Myc in Glioma Cells Stimulate Glioma Cell Migration and Suppresses Glioma Cell Proliferation *in vitro*

**Figure 7:**
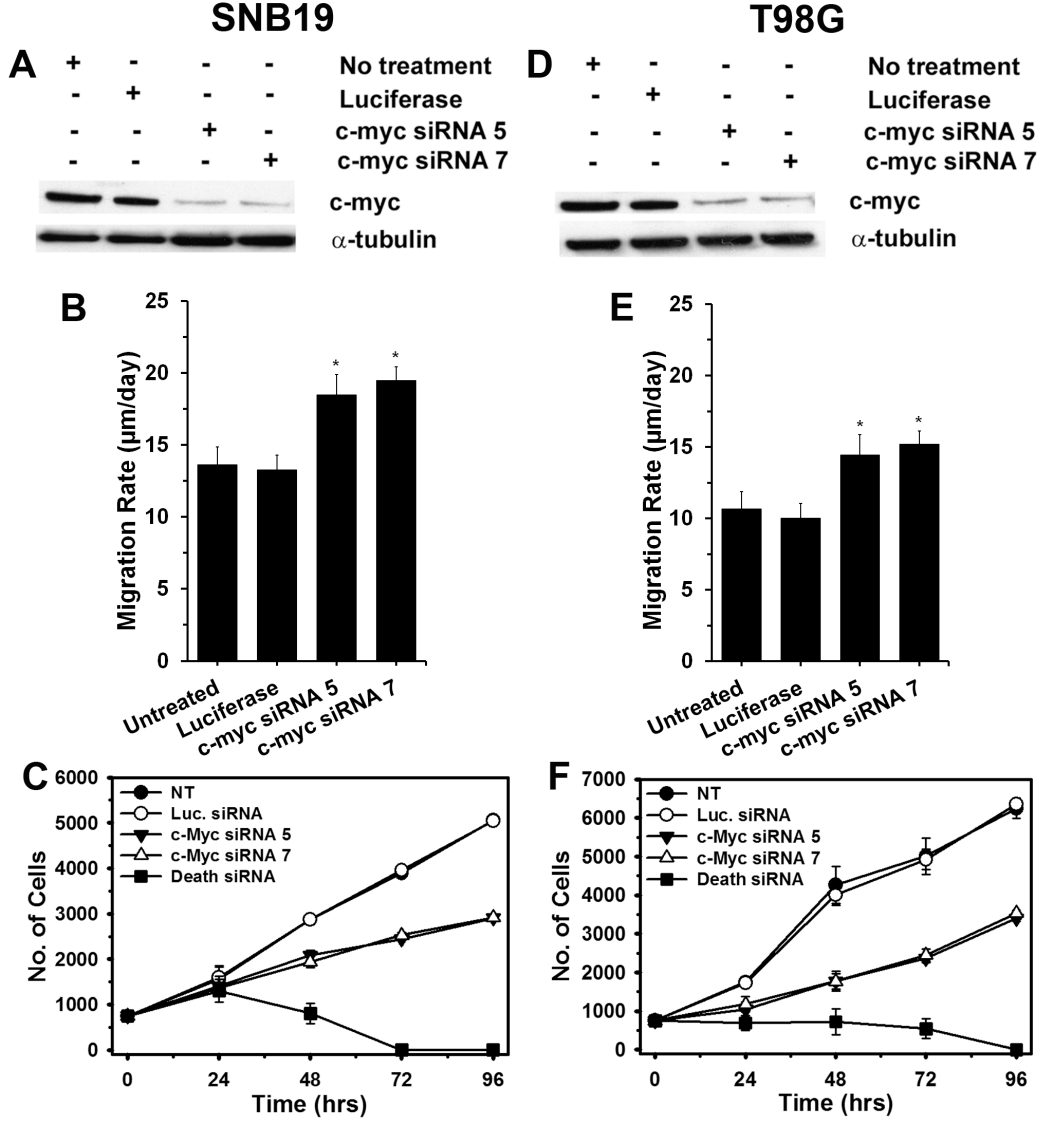
Knockdown of c-Myc using siRNA increases glioma cell migration and reduces glioma cell proliferation. Western blot analysis (α-tubulin used as a loading control) to confirm reduction of c-Myc in the glioma cell lines (A) SNB19 and (D) T98G transfected with two c-Myc–specific siRNA. Untransfected cells and cells transfected with a control siRNA (luciferase) are shown for comparison. Treatment with two separate c-Myc siRNA increased migration of (B) SNB19 and (E) T98G cells in a radial migration assay when compared with untreated or luciferase-transfected cells (p<0.001, 2-tailed Student’s t-test). Treatment with two c-Myc siRNA suppressed proliferation of (C) SNB19 and (E) T98G as demonstrated by alamar blue assay when compared with untreated or luciferase transfected cells. Cell Death siRNA (Qiagen) was utilized in the proliferation assay as internal experimental control.

To examine the functional roles of c-Myc, two independent sequences of siRNA designed specifically to inhibit c-Myc were transfected into SNB19 and T98G glioma cells. Western blot analysis showed >90% decrease of c-Myc protein levels in both cell lines transfected with the two c-Myc siRNA as compared to control siRNA (Figure 7A & D). Monolayer radial migration assays revealed that depletion of c-Myc expression by siRNA oligonucleotides in SNB19 and T98G glioma cells significantly increased migration rates relative to siRNA control transfection or untreated cells (p<0.001) (Figure 7, B & E). A cell proliferation assay demonstrated that inhibition of c-Myc in SNB19 and T98G glioma cells suppressed cell proliferation as compared to mock transfected and untreated cells (Figure 7, C & F). Cell counts in experiments where c-Myc was knocked down differed substantially from those where a cocktail of siRNAs fatal to cells was used.

### Inhibition of Canonical NF-κB Activity Suppresses Glioma Cell Migration and Invasion

**Figure 8:**
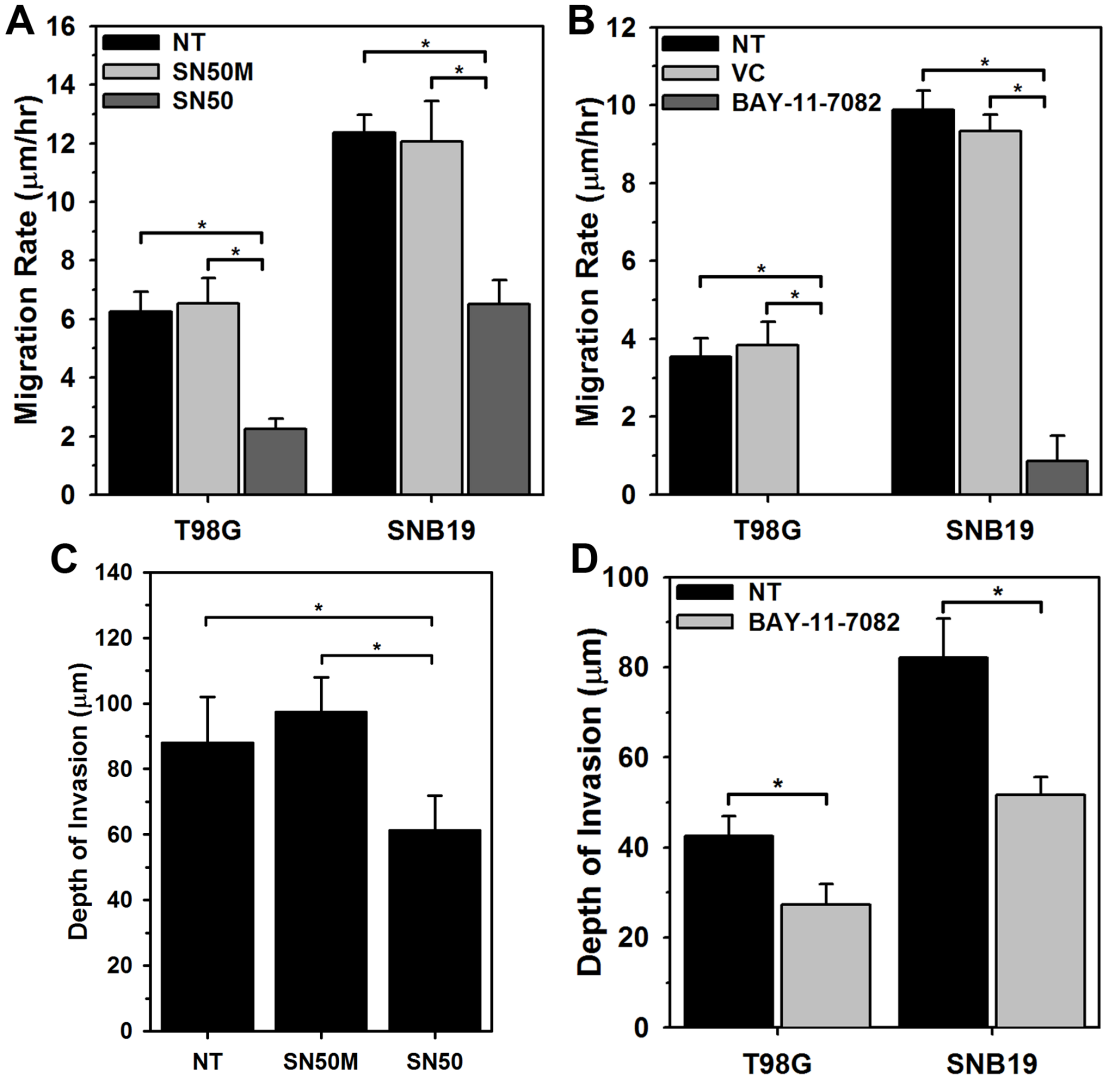
Inhibition of NF-κB function suppresses glioma cell migration in vitro and invasion ex vivo. Treatment with 50 µM SN50 peptide inhibitor suppresses migration of (A) T98G and SNB19 glioma cells in a radial migration assay when compared with untreated (NT) or scrambled peptide treated (SN50M) cells (*; p<0.0001). (B) SNB19 glioma cells stably expressing GFP were implanted into the bilateral putamen on rat organotypic brain slices. Implanted cells were then treated with SN50 peptide inhibitor, scrambled peptide (SN50M) or left untreated (NT) and observed at 48 hrs. Depth of invasion was then calculated from Z-axis images collected by confocal laser scanning microscopy. The mean value of the depth of invasion was obtained from four independent experiments (*; p<0.0001). Treatment with 20 µM NF-κB functional inhibitor (BAY-11-7082) suppresses migration of (C) T98G and SNB19 glioma cells in a radial migration assay when compared with untreated (NT) or DMSO treated (VC) cells (*; p<0.0001). (D) Similar ex vivo brain slice invasion assay as described in B with NF-κB functional inhibitor (BAY-11-7082) demonstrated reduced invasion of T98G and SNB19 glioma cells. The mean value of the depth of invasion was obtained from four independent experiments (*; p<0.0001).

To examine the functional role of NF-κB, T98G and SNB19 glioma cell lines were treated with two different NF-κB inhibitors: a cell-permeable peptide inhibitor, SN50, and a small molecule inhibitor BAY-11-7082. SN50 peptide consists of an N-terminal hydrophobic peptide fused to the p50 nuclear localization sequence. SN50 inhibits NF-κB activity by inhibiting p50/RelA (p65) nuclear translocation [40]. This peptide has no effect on the transcriptional activities of other factors such as AP-1 and CREB [41]. Glioma cells treated with the SN50 peptide inhibitor displayed ∼50–60% decrease in cell migration (p<0.0001; Figure 8A). Analysis of the cytological location of the p65 subunit of NF-κB on the fixed migrated cells (immunofluorescent for p65) showed retention of p65 protein in the cytoplasm (data not shown). In comparison, cell-permeable inactive control peptide, SN50M, had no effect on glioma cell migration. No toxicity was observed in cells treated with SN50 (data not shown), further suggesting that the effect of cell motility is not due to cell death. Furthermore, the effect of NF-κB inhibition on glioma cell invasion was examined using an *ex vivo* organotypic rat brain slice model [31], [42]. Invasion of SNB19-GFP-expressing human glioma cells into the rat brain slices over 48 hours was quantified by confocal microscopy. Inhibition of NF-κB resulted in a ∼30% decrease in cell invasion of SNB19 cells as compared to untreated cells or cells treated with SN50M (p<0.0001, Figure 8B). This data corroborate the cell migration results and further support a role NF-κB in glioma cell migration and invasion.

The small molecule inhibitor BAY-11-7082 selectively and irreversible inhibits NF-κB activation by blocking cytokine induced phosphorylation of IκBα without affecting constitutive IκBα phosphorylation [43]. Radial migration assay demonstrated that functional inhibition of NF-κB using the BAY-11-7082 in glioma cells reduces cell migration by ∼85-100% (P<0.0001: Figure 8C) relative to vehicle (DMSO) treated or untreated cells. Very low cytotoxicity (∼10%) was observed in cells treated with BAY-11-7082 (data not shown), suggesting that the extreme effect of cell motility is not solely due to cell death. Further, BAY-11-7082 also demonstrated 40 - 45% reduction in cell invasion of SNB19 and T98G cells as compared to untreated cells (p<0.0001, Figure 8C). These data suggest that activation of NF-κB is important for glioma cell migration.

## Discussion

In our previous work we found that the gene expression profile of invading glioma cells reveals a pattern unique to this discrete population of cells when compared to the gene expression profile of stationary glioma cells [14]. In addition, invading glioma cells show higher expression of anti-apoptotic and motility related genes [14]. In this work, we report that glioma cells at the core of the tumor have higher levels of Ki-67 mRNA expression (Figure 1) as compared to the glioma cells at the invading rim, suggesting an enhanced tendency to proliferate by cells at the core or a diminished proliferative commitment by cells that are invading. Further, immunohistochemical validation using a brain tumor TMA portrays the highest level of Ki-67 protein expression in GBM cells at the stationary core rather than at the invading rim (Figure 2). *In vitro* BrdU incorporation and radial migration experiments showed that cells committed to cell cycle have a lower tendency to migrate, while non-proliferative cells have increased propensity to migrate (Figure 3).

Transcription factor profiling indicated differential activation of multiple transcription factors between cells at the core (migration restricted) and the rim (migration stimulated) (Figure 4), and it may be possible that phenotypic behavior of glioma cells is choreographed by activation and/or suppression of multiple transcription factors. Testing the functional consequence of interfering with transcription factors whose activation occurs during migration or proliferation, such as NF-κB and c-Myc may be a strategy by which to identify those transcription factors that are master regulators of phenotypic shifts in glioma cells. Additionally, analysis of the invasion microarray dataset implicated regulation of IκB/NF-κB cascade as a recurring ontology with high statistical significance (data not shown) corroborating with the transcription factor analysis of migrating glioma cells. Conversely, migration-restricted cells show highest activation of c-Myc in all four glioma cell lines, which is known to be a key transcription factor in regulating cell cycle [44], [45]; which corroborates findings from our previous experiments. Further, differential activation of c-Myc and NF- κB in glioma cells at the core and the rim was also observed in radial cell migration assay *in vitro* (Figure S6). Importantly, immunohistochemical validation using a malignant glioma TMA revealed the highest level of phospho-c-Myc protein in GBM cells at the stationary core and highest level of activated NF-κB protein expression in GBM cells at the invading rim (Figure 5). These findings were further validated *in vitro* with radial migration assay using engineered c-Myc and NF-κB reporter cell lines. That assay demonstrated higher expression of tdTomato protein linked to c-Myc activation in the core as compared to the rim and higher expression GFP protein linked to NF-κB activation in the rim as compared to the core (Figure 6). Consequently, we investigated the functional role of NF-κB and c-Myc in orchastrating phenotypic behaviors of glioma cells at the stationary core and the invading rim of GBM patient tumor cases.

Our results testing 19 transcription factors show that NF-κB and c-Myc are highly activated in migration-stimulated and migration-restricted GBM cells, respectively. Other studies establish critical roles for NF-κB and c-Myc separately in different types of cancers [46], [47]. However, to our knowledge, this is the first study that links phenotypic status of glioma cells to the differential activation of these two transcription factors. Genomic and functional analyses of c-Myc targets suggest that c-Myc aids in expression of genes involved in cell cycle regulation, apoptosis, metabolism, ribosome biogenesis, protein synthesis, and mitochondrial function, while c-Myc also consistently represses genes involved in cell growth arrest and cell adhesion [46], [48]. In support of these previous findings we show that when c-Myc is depleted, glioma cells switch their proliferative phenotype to a migratory phenotype accompanied by a decrease in cell proliferation (Figure 7). This observation affirms that cell proliferation and migration are two associated but dichotomous processes and cells have lesser tendency to migrate when they are proliferating. On the other hand, there is compelling evidence that transcription factor NF-κB aids in expression of genes involved in cell survival, invasion/metastases, and inflammation [49]. And as expected when NF-κB function was inhibited using the pharmacological inhibitor BAY-11-7082, glioma cells demonstrated reduced migration and invasion (Figure 8). However, reduction in migration was accompanied by reduced or unchanged rates of proliferation (Figure S7), which deviates from the hypothesis that cell proliferation and migration are two associated but dichotomous processes. This contradictory cell behavior may be attributed to the disappearance of cell survival signals associated with NF-κB or sustained signaling from other transcription factors including AP2, GATA or JAK/STAT proteins. Recently, Zanotto-Filho *et al* demonstrated that pharmacological inhibition of NF-κB using a variety of different small molecule inhibitors and down-regulation of NF-κB using siRNA leads to cell apoptosis [50]. Romashkova and co-workers have demonstrated that NF-κB signaling is necessary to negate cytotoxic effect of c-Myc and to maintain cell proliferation via growth factor signaling [51]. A review by Larsson *et al* describes a *yin-yang* function of c-Myc oncoprotein in cancer and how c-Myc promotes intrinsic tumor suppressor mechanisms including apoptosis, cellular senescence, and DNA damage response [52]. Previous findings, as well as our results reported here, suggest that some basal expression level of NF-κB is necessary to maintain the proliferative phenotype of glioma cells; it is possible that the phenotypic fate of glioma cells is dictated by the ratio of proliferative vs migratory transcription factor functions, such as c-Myc and NF-κB. This interpretation points to a very decisive role of the tumor microenvironment and associated extracellular signaling, which govern the activity of transcription factors, in conveying phenotypic behavior to glioma cells.

The transcription factor NF-κB plays important roles in a myriad of physiological and pathological phenotypes [53]. NF-κB can regulate over 250 genes and a subset of these genes is linked to cancer cell invasion and survival [54]. However, it is unclear whether NF-κB targeted genes are differentially expressed in subpopulations of cells actively invading or proliferating in the localized tumor core. To address this question, we mined the expression levels of ∼150 known NF-κB target genes in the transcriptional profiling of laser capture-microdissected invasive GBM cells. The candidate genes are grouped accordingly to their known function in relation to cancer biology and progression (Figure S8). NF-κB targeted genes encoding transcription factors and functioning in cell proliferation (i.e. MYC, MYB, RELB, S100A4, S100A10) are highly expressed in the core of the majority of GBM specimens. Additionally, genes encoding growth factors and their downstream modulators (e.g. VEGFA, IGFBP2) are increased in the core of GBM specimens as compared to the corresponding rim cells. In contrast, the expression levels of NF-κB regulated genes encoding anti-apoptotic proteins (e.g. BCL-XL, ENC1) are more highly expressed in the invasive rim subpopulation of GBM specimens as compared to their matched tumor core. Interestingly, certain cell surface receptors such as EGFR and ABCB1 (a membrane-associate protein involved in multidrug resistance) are also highly expressed in invading cells. These data suggest that NF-κB targeted genes are differentially expressed between the subpopulation of invasive GBM cells and cells residing in the tumor core. Further, functions of NF-κB regulated genes correspond well with observed phenotypic behavior of glioma cells at the core and rim.

The transcription factor c-Myc is one of the most frequently activated oncogenes and is estimated to be involved in 20% of all human cancers [55], [56]. The c-Myc target gene network is estimated to be comprised of about 15% of all genes and a subset of these genes is associated with regulation of cell cycle and apoptosis [46]. However, it is unclear whether c-Myc regulated genes are differentially expressed in subpopulations of cells actively invading or proliferating in the localized tumor core. To address this question, we mined the expression levels of ∼400 (www.myccancergene.org) known c-Myc target genes in the transcriptional profiling of laser capture-microdissected invasive GBM cells. The candidate genes are grouped accordingly to their known function in relation to cancer biology and progression (Figure S9). c-Myc targeted genes functioning in regulating cell cycle and which are necessary for active cell proliferation (e.g. CCNA2, CCNB1, CCND1, CDK1, CDK4, CDK6) are highly expressed in the core of the majority of the GBM specimen. Conversely, genes encoding proteins involved in cell growth arrest (i.e. DDIT3, MT3, SYK) are expressed in the invasive rim subpopulations of GBM specimens as compared to their matched tumor core. Interestingly, genes encoding pro-apoptotic proteins (i.e. CASP3, NOD1, BARD1) are more expressed in the core of the majority of GBM specimens. In contrast, the expression level of c-Myc target genes encoding for anti-apoptotic proteins (i.e. CRYAB, MCL1) are highly expressed in the invasive rim subpopulation. Additionally, c-Myc regulated genes encoding transcription factors and functioning in cell proliferation and apoptosis (i.e. E2F1, E2F2, BCL3, HIF1A, MYCBP, FOXM1) are highly expressed in the core of the majority of GBM specimens. c-Myc targeted genes encoding proteins involved in cell migration and cell invasion (i.e. TGF1, TGF2, SPAC, TAX-1, PRDX5, MAP2K5) are increased in the rim of GBM specimens as compared to the corresponding core cells. In contrast, the expression levels of c-Myc target genes encoding proteins involved in cell proliferation (i.e. EMP1, HMMR) are more expressed in the stationary core subpopulations of GBM specimens as compared to their matched tumor rim. These data suggest that c-Myc regulated genes are differentially expressed between the subpopulation of invasive GBM cells and cells residing in the tumor core. Further, functions of c-Myc regulated genes correspond well with observed phenotypic behavior of glioma cells at the core and rim.

In summary, these findings portray that invasive, dispersive glioblastoma cells are distinct from their cognate, stationary partners in terms of phenotype (invasive versus proliferative; “go versus grow”). The results posit reciprocal activation of two transcription factors, NF-κB and c-Myc, acting alone or with other transcription factors so as to drive the expression of specific genes encoding proteins whose functions drive cell invasion and survival in contradistinction to cell proliferation, respectively. Interactions between GBM cells and the immediate micro-anatomical niche are likely to be dynamic and mutually responsive in such a way that nurtures and promotes adaptive quiescence, growth, invasion, and progression of GBM. The differential expression (upregulation) of cell survival genes in the invasive GBM population of cells warrants creative and specific targeted therapeutic intervention to control this most troublesome of the tumor cells left behind after surgery.

## Supporting Information

Figure S1 Fluorescent micrographs (x10 magnification) of matched (A) core and (B) rim regions on glioma TMA immunofluorescently stained with a monoclonal antibody against Ki-67 and analyzed by a HistoRx imaging system. Red, Ki-67 staining; green, GFAP for glial fiber staining; blue, DAPI nuclear staining.
(TIF)

Figure S2 Glioma cells seeded in a way that manifests cell crowding and cell dispersion show that at the core cells were more proliferative than glioma cells located at the rim. (A) SNB19 cells at the core of the cell circle stained for CyclinA (Cy3-Red). (B) Same image field as panel A at the core of the cell circle but stained for incorporated BrdU (FITC-green). (C) Same image field as panel A and B at the core of the cell circle but showing CyclinA (Cy3-Red) - BrdU (FITC-green) Overlay. (D) SNB19 cells at the rim of the cell circle stained for CyclinA (Cy3-Red). (E) Same image field as panel D at the rim of the cell circle but stained for incorporated BrdU (FITC-green). (F) Same image field as panel D and E at the rim of the cell circle but showing CyclinA (Cy3-Red) - BrdU (FITC-green) Overlay.
(TIF)

Figure S3 Transcription Factor Profiling of Migrating Cancer Cells vs Migration-Restricted Cancer Cells. Glioma cells were seeded on glioma-derived ECM or non-glioma tumor cells were seeded on collagen type IV under migration-activated “sparse” or in migration-restricted “dense” condition. Two independent biological replicates were performed with each sample in triplicate. Ratios of the averaged mean fluorescent intensities for each transcription factor for sparse over dense were calculated for each biological set and are plotted in the heat map using a conditionally formatted color range. Green boxes represent the transcription factors activated when cells were in a migration-activated condition (sparse/dense ratios ≥1.5). Red boxes represent transcription factors activated when cells were in a migration-restricted condition (sparse/dense ratios ≤0.6). Yellow boxes indicate no change in transcription activity (sparse/dense ratios between 0.65 and 1.5).
(TIF)

Figure S4 Glioma tumor specimens show differential activation of c-Myc and NFκB in core and invasive rim. Immunohistochemistry of glioma sample showing core and rim of the tumor in the same field of view for comparision. (A) Phosphorylated c-Myc nuclear protein expression is greater at the glioma tumor core (Indicated by C) than the rim (indicated by R) regions of tumor. (B) Phosphorylated NFκB nuclear protein expression is greater at the glioma tumor rim (Indicated by R) than the core regions of tumor. Black arrows represent the invading glioma tumor cells staned negatively for Phospho c-Myc and positively for Phospho NFκB.
(TIF)

Figure S5 Migrating glioma cells promote activation of the transcription factor NF-κB whereas migration-restricted glioma cells display high c-Myc activation. T98G and SNB19 glioma cells were infected with lentivirus expressing the binding element for either the transcription factor NFkB and a green fluorescent protein (GFP) reporter or the transcription factor c-Myc and a red fluorescent protein (tdTomato) reporter. Higher magnification fluorescent micrographs (40X) of mCMV control GFP vector, NF-κB GFP reporter vector, control tdTomato vector, and tdTomato c-Myc reporter vector infected T98G and SNB19 glioma cells. Green cells are GFP positive and blue cells are not expressing the GFP protein but are stained with Hoescht stain. Red cells are tdTomato positive and blue cells are not expressing the tdTomato protein but are stained with Hoescht stain. Fluorescent micrographs of the core and the corresponding rim regions are shown in the micrographs.
(TIF)

Figure S6 Glioma cells at the rim in a migratory setting demonstrate higher activation of NF-κB than glioma cells at the core and glioma cells at the core in a migratory setting demonstrate higher activation of c-Myc than glioma cells at the rim. (A) T98G cells at the core of the cell circle stained for DAPI to account for all cells. (B) Same image field as panel A at the core of the cell circle but stained with phospho NF-κB (Cy3-red). (C) Merged image from panels A and B. (D) T98G cells at the rim of the cell circle stained for DAPI to account for all cells. (E) Same image field as panel D but stained with phospho NF-κB (Cy3-red). (F) Merged image from panels D and E. (G) T98G cells at the core of the cell circle stained for DAPI to account for all cells. (H) Same image field as panel A at the core of the cell circle but stained with phospho c-Myc (Cy3-red). (I) Merged image from panels G and H. (J) T98G cells at the rim of the cell circle stained for DAPI to account for all cells. (K) Same image field as panel D but stained with phospho c-Myc (Cy3-red). (L) Merged image from panels J and K.
(TIF)

Figure S7 Treatment with pharmacological inhibitor of NF-κB, BAY-11-7082, do not change proliferation of (A) T98G and suppresses proliferation of (B) SNB19 as demonstrated by alamar blue assay when compared with untreated or DMSO treated (VC) cells. Cell were treated with 20 µM BAY-11-7082 at 24 hr time.
(TIF)

Figure S8 Differential expression of NF-κB targeted genes in core and invasive rim of GBM specimens. In silico analysis of NF-κB-targeted genes were performed on the transcriptional profiling database of laser capture-microdissected cells collected form paired patient GBM tumors core and invading rim (N = 19). The relative ratio of rim to core mRNA expression for each NF-κB target genes were compared for 19 specimens and candidate genes were selected where probes were defined as differentially expressed by a 2 fold difference between core and rim samples with a p-value cutoff of 0.05. The known candidate NF-κB targeted genes whose expression values where differentially expressed between core or rim samples in 9+ GBM specimens are listed according to biological functions.
(TIF)

Figure S9 Differential expression of c-Myc targeted genes in core and invasive rim of GBM specimens. In silico analysis of c-Myc-targeted genes were performed on the transcriptional profiling database of laser capture-microdissected cells collected form paired patient GBM tumors core and invading rim (N = 19). The relative ratio of rim to core mRNA expression for each c-Myc target genes were compared for 19 specimens and candidate genes were selected where probes were defined as differentially expressed by a 2 fold difference between core and rim samples with a p-value cutoff of 0.05. The known candidate c-Myc targeted genes whose expression values where differentially expressed between core or rim samples in 9+ GBM specimens are listed according to biological functions.
(TIF)
