# Supplementary figures and images for: Reciprocal Activation of Transcription Factors Underlies the Dichotomy between Proliferation and Invasion of Glioma Cells
Source: PLoS One. 2013 Aug 15;8(8):e72134. doi: 10.1371/journal.pone.0072134 (PMC3744529; doi:10.1371/journal.pone.0072134)

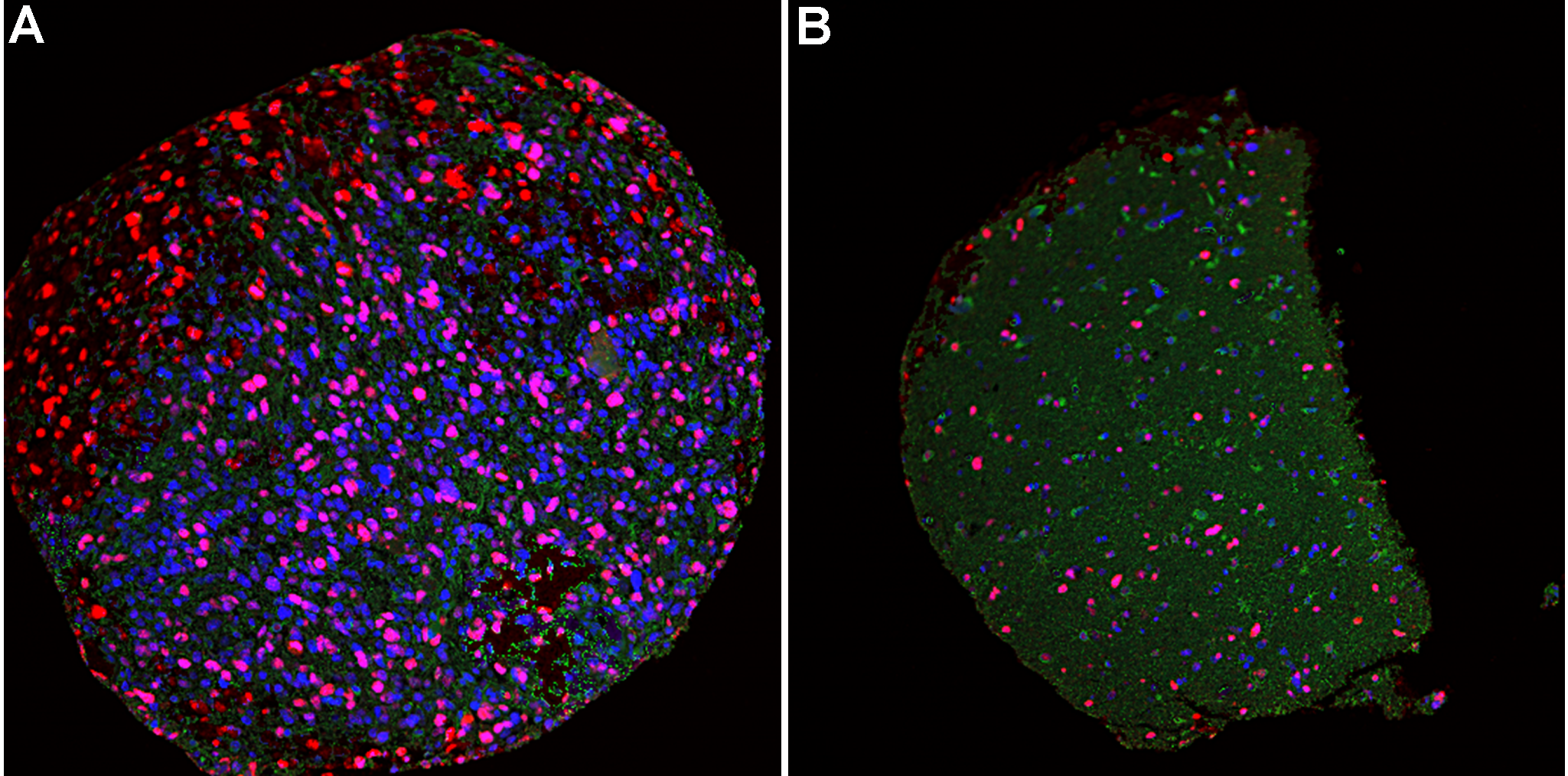

Supplement: Figure S1 — Fluorescent micrographs (x10 magnification) of matched (A) core and (B) rim regions on glioma TMA immunofluorescently stained with a monoclonal antibody against Ki-67 and analyzed by a HistoRx imaging system. Red, Ki-67 staining; green, GFAP for glial fiber staining; blue, DAPI nuclear staining. (TIF) [file pone.0072134.s001.tif]

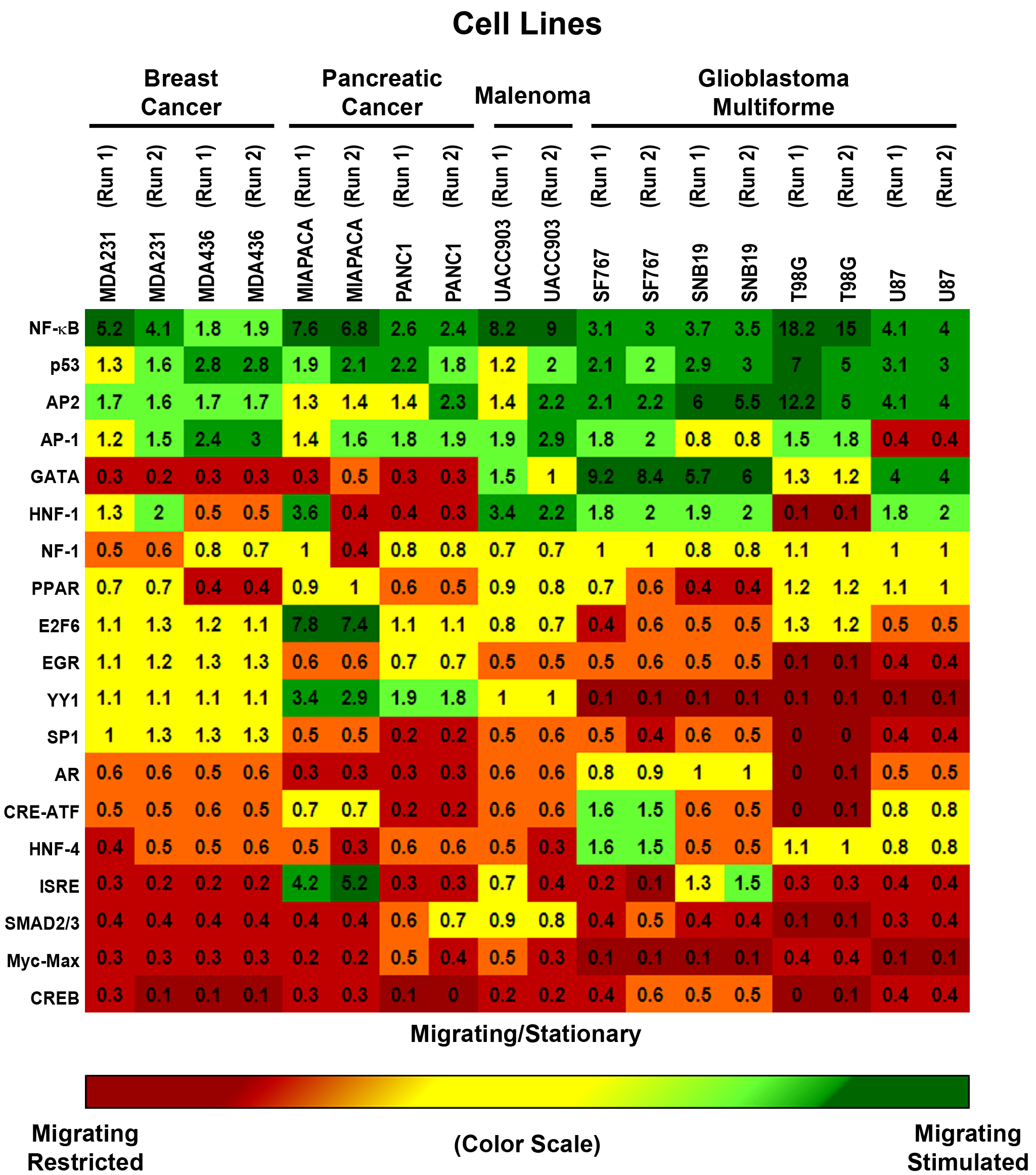

Supplement: Figure S2 — Glioma cells seeded in a way that manifests cell crowding and cell dispersion show that at the core cells were more proliferative than glioma cells located at the rim. (A) SNB19 cells at the core of the cell circle stained for CyclinA (Cy3-Red). (B) Same image field as panel A at the core of the cell circle but stained for incorporated BrdU (FITC-green). (C) Same image field as panel A and B at the core of the cell circle but showing CyclinA (Cy3-Red) - BrdU (FITC-green) Overlay. (D) SNB19 cells at the rim of the cell circle stained for CyclinA (Cy3-Red). (E) Same image field as panel D at the rim of the cell circle but stained for incorporated BrdU (FITC-green). (F) Same image field as panel D and E at the rim of the cell circle but showing CyclinA (Cy3-Red) - BrdU (FITC-green) Overlay. (TIF) [file pone.0072134.s002.tif]

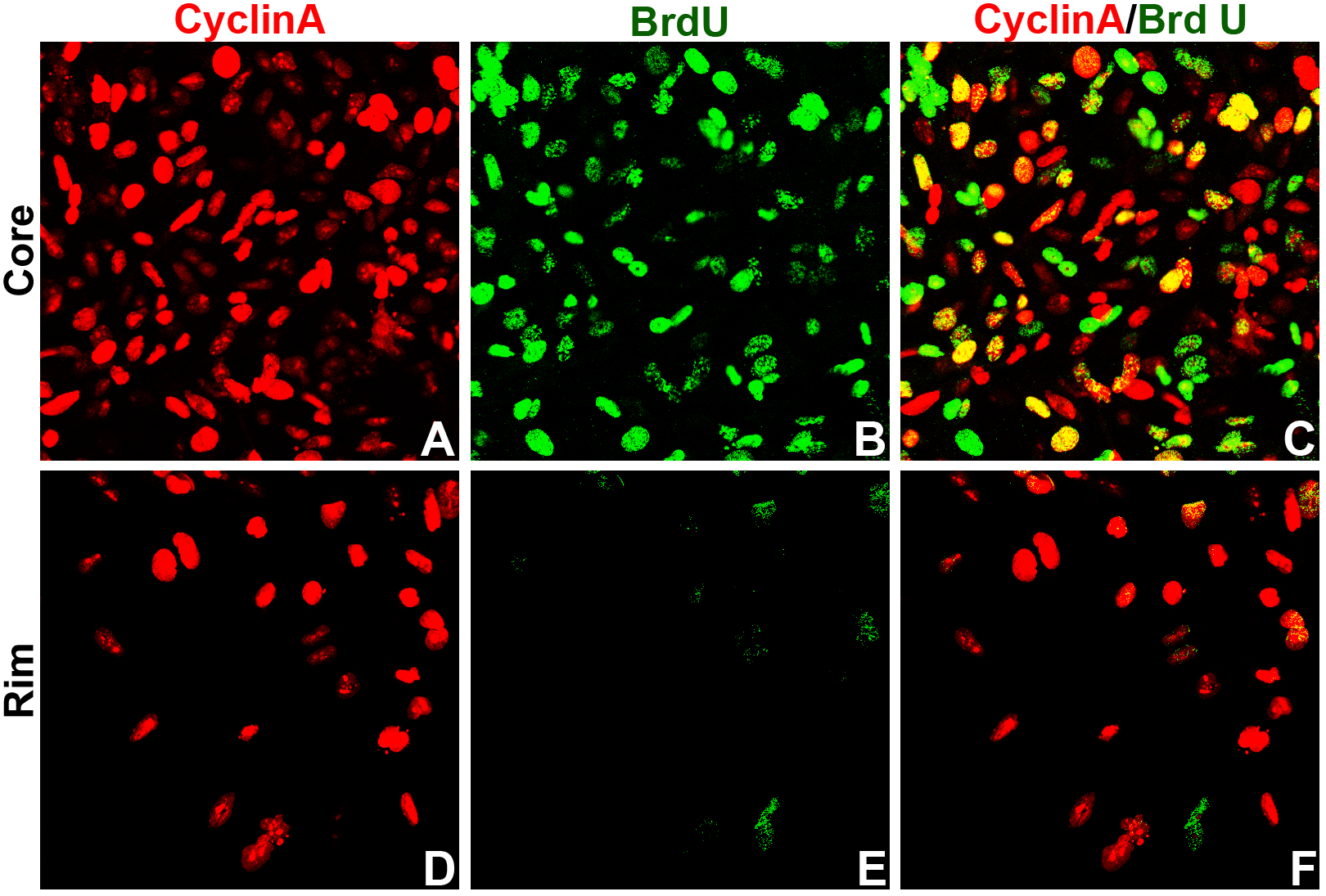

Supplement: Figure S3 — Transcription Factor Profiling of Migrating Cancer Cells vs Migration-Restricted Cancer Cells. Glioma cells were seeded on glioma-derived ECM or non-glioma tumor cells were seeded on collagen type IV under migration-activated “sparse” or in migration-restricted “dense” condition. Two independent biological replicates were performed with each sample in triplicate. Ratios of the averaged mean fluorescent intensities for each transcription factor for sparse over dense were calculated for each biological set and are plotted in the heat map using a conditionally formatted color range. Green boxes represent the transcription factors activated when cells were in a migration-activated condition (sparse/dense ratios ≥1.5). Red boxes represent transcription factors activated when cells were in a migration-restricted condition (sparse/dense ratios ≤0.6). Yellow boxes indicate no change in transcription activity (sparse/dense ratios between 0.65 and 1.5). (TIF) [file pone.0072134.s003.tif]

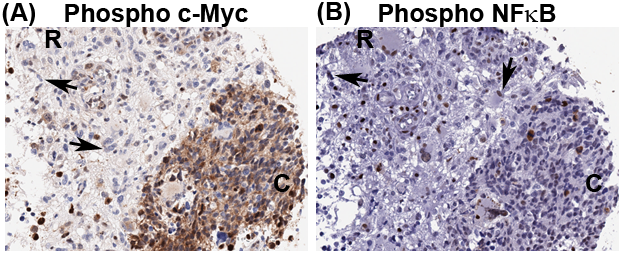

Supplement: Figure S4 — Glioma tumor specimens show differential activation of c-Myc and NFκB in core and invasive rim. Immunohistochemistry of glioma sample showing core and rim of the tumor in the same field of view for comparision. (A) Phosphorylated c-Myc nuclear protein expression is greater at the glioma tumor core (Indicated by C) than the rim (indicated by R) regions of tumor. (B) Phosphorylated NFκB nuclear protein expression is greater at the glioma tumor rim (Indicated by R) than the core regions of tumor. Black arrows represent the invading glioma tumor cells staned negatively for Phospho c-Myc and positively for Phospho NFκB. (TIF) [file pone.0072134.s004.tif]

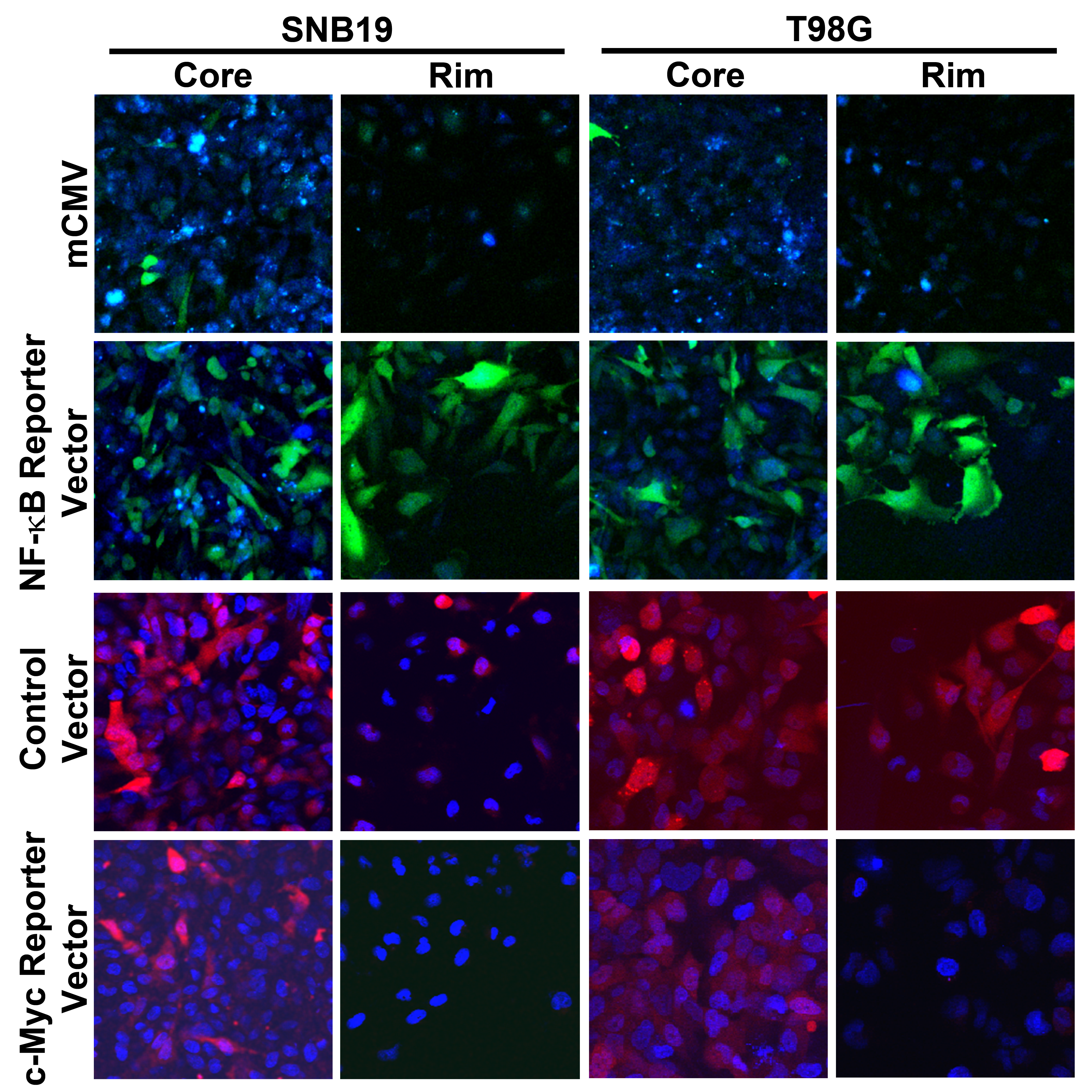

Supplement: Figure S5 — Migrating glioma cells promote activation of the transcription factor NF-κB whereas migration-restricted glioma cells display high c-Myc activation. T98G and SNB19 glioma cells were infected with lentivirus expressing the binding element for either the transcription factor NFkB and a green fluorescent protein (GFP) reporter or the transcription factor c-Myc and a red fluorescent protein (tdTomato) reporter. Higher magnification fluorescent micrographs (40X) of mCMV control GFP vector, NF-κB GFP reporter vector, control tdTomato vector, and tdTomato c-Myc reporter vector infected T98G and SNB19 glioma cells. Green cells are GFP positive and blue cells are not expressing the GFP protein but are stained with Hoescht stain. Red cells are tdTomato positive and blue cells are not expressing the tdTomato protein but are stained with Hoescht stain. Fluorescent micrographs of the core and the corresponding rim regions are shown in the micrographs. (TIF) [file pone.0072134.s005.tif]

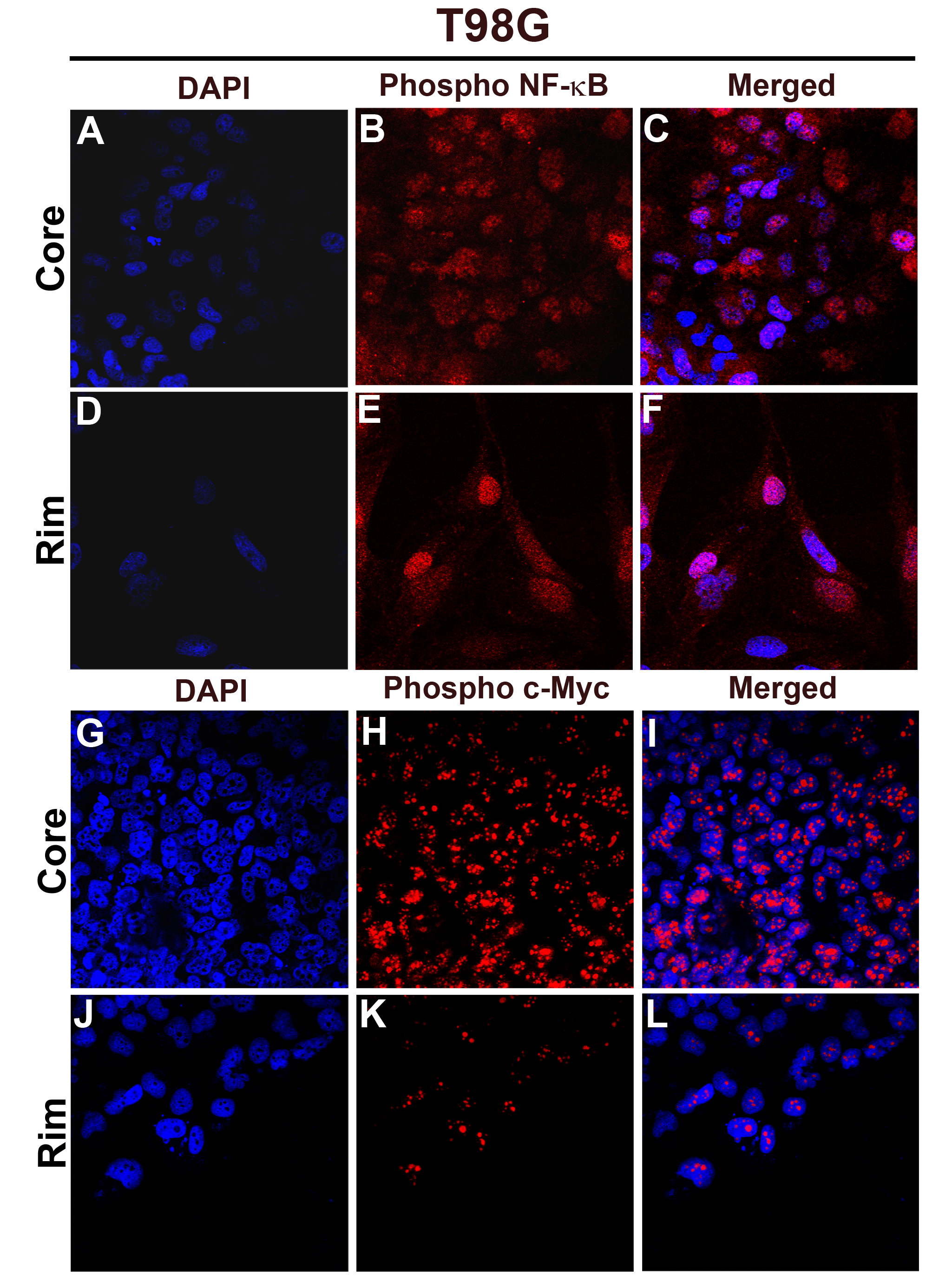

Supplement: Figure S6 — Glioma cells at the rim in a migratory setting demonstrate higher activation of NF-κB than glioma cells at the core and glioma cells at the core in a migratory setting demonstrate higher activation of c-Myc than glioma cells at the rim. (A) T98G cells at the core of the cell circle stained for DAPI to account for all cells. (B) Same image field as panel A at the core of the cell circle but stained with phospho NF-κB (Cy3-red). (C) Merged image from panels A and B. (D) T98G cells at the rim of the cell circle stained for DAPI to account for all cells. (E) Same image field as panel D but stained with phospho NF-κB (Cy3-red). (F) Merged image from panels D and E. (G) T98G cells at the core of the cell circle stained for DAPI to account for all cells. (H) Same image field as panel A at the core of the cell circle but stained with phospho c-Myc (Cy3-red). (I) Merged image from panels G and H. (J) T98G cells at the rim of the cell circle stained for DAPI to account for all cells. (K) Same image field as panel D but stained with phospho c-Myc (Cy3-red). (L) Merged image from panels J and K. (TIF) [file pone.0072134.s006.tif]

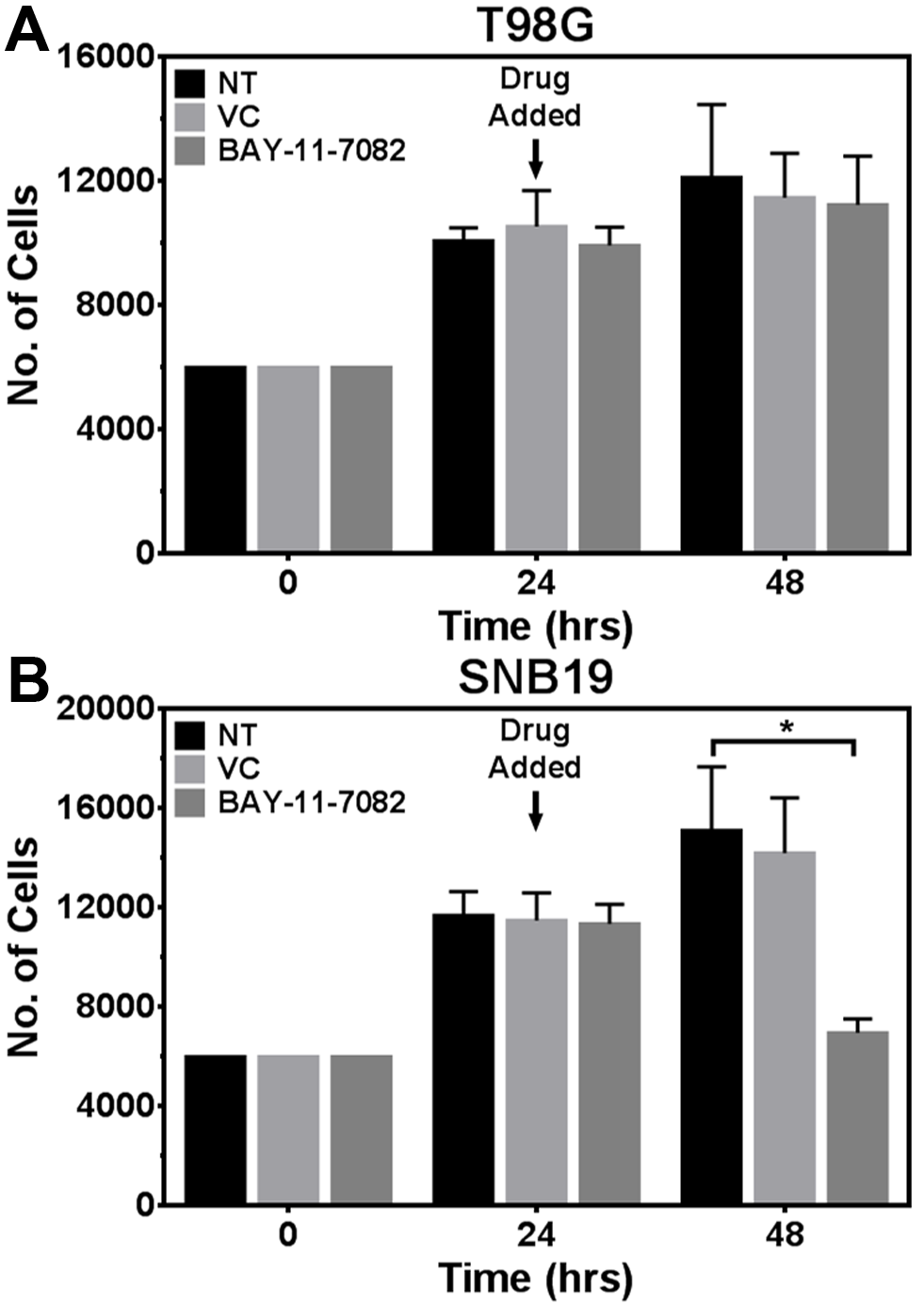

Supplement: Figure S7 — Treatment with pharmacological inhibitor of NF-κB, BAY-11-7082, do not change proliferation of (A) T98G and suppresses proliferation of (B) SNB19 as demonstrated by alamar blue assay when compared with untreated or DMSO treated (VC) cells. Cell were treated with 20 µM BAY-11-7082 at 24 hr time. (TIF) [file pone.0072134.s007.tif]

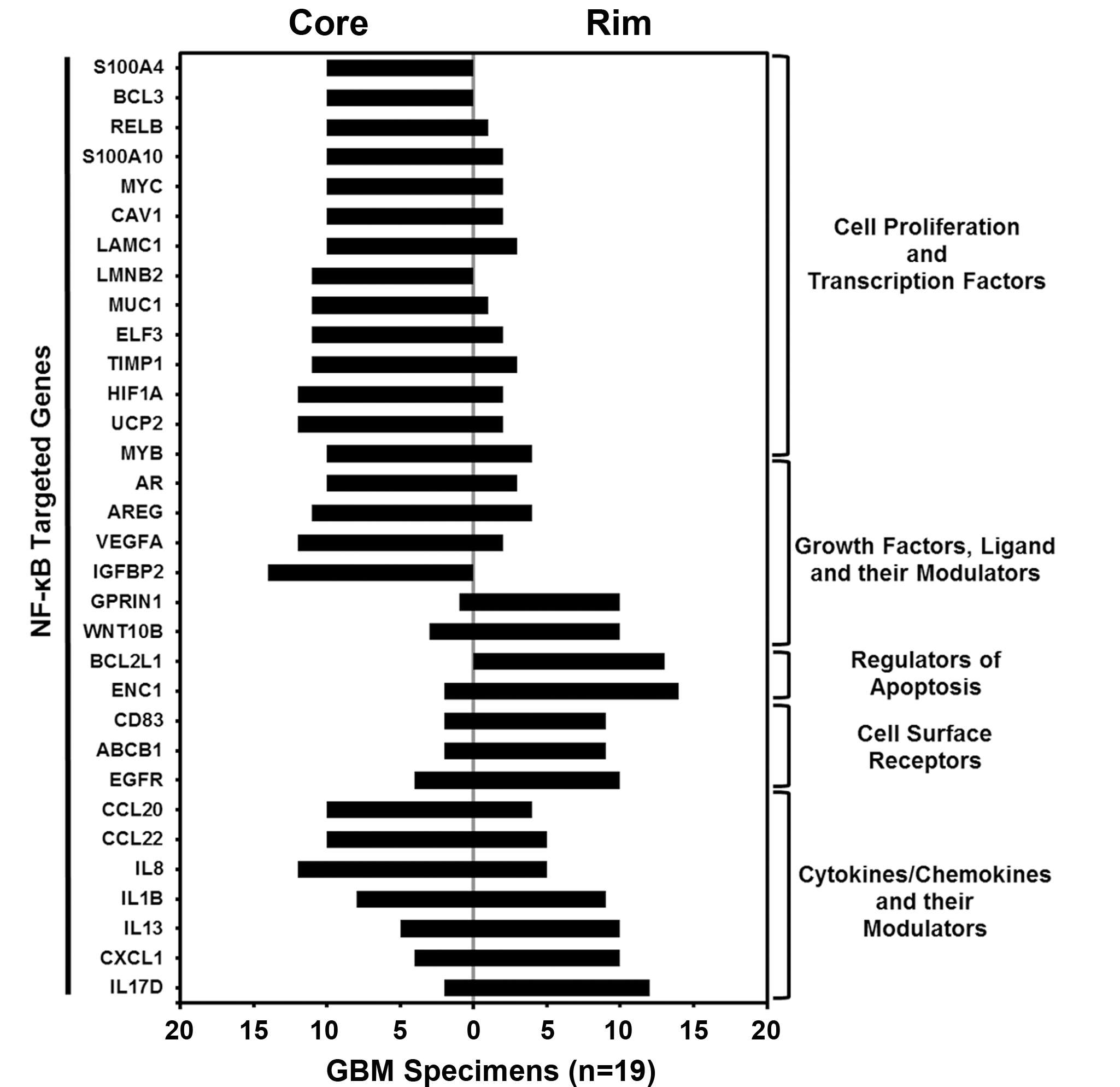

Supplement: Figure S8 — Differential expression of NF-κB targeted genes in core and invasive rim of GBM specimens. In silico analysis of NF-κB-targeted genes were performed on the transcriptional profiling database of laser capture-microdissected cells collected form paired patient GBM tumors core and invading rim (N = 19). The relative ratio of rim to core mRNA expression for each NF-κB target genes were compared for 19 specimens and candidate genes were selected where probes were defined as differentially expressed by a 2 fold difference between core and rim samples with a p-value cutoff of 0.05. The known candidate NF-κB targeted genes whose expression values where differentially expressed between core or rim samples in 9+ GBM specimens are listed according to biological functions. (TIF) [file pone.0072134.s008.tif]

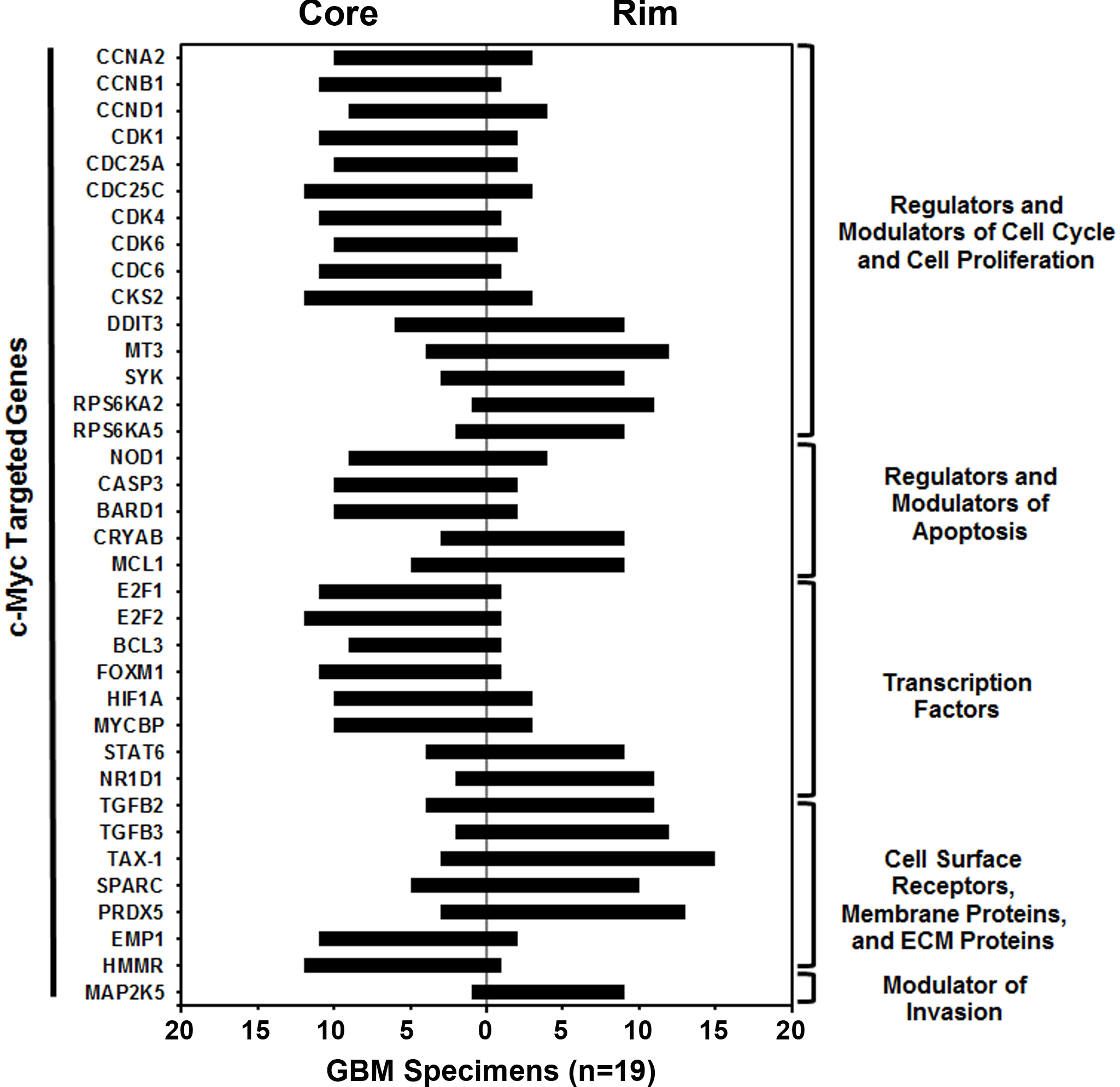

Supplement: Figure S9 — Differential expression of c-Myc targeted genes in core and invasive rim of GBM specimens. In silico analysis of c-Myc-targeted genes were performed on the transcriptional profiling database of laser capture-microdissected cells collected form paired patient GBM tumors core and invading rim (N = 19). The relative ratio of rim to core mRNA expression for each c-Myc target genes were compared for 19 specimens and candidate genes were selected where probes were defined as differentially expressed by a 2 fold difference between core and rim samples with a p-value cutoff of 0.05. The known candidate c-Myc targeted genes whose expression values where differentially expressed between core or rim samples in 9+ GBM specimens are listed according to biological functions. (TIF) [file pone.0072134.s009.tif]
